# Adaptive Evolution and Environmental Durability Jointly Structure Phylodynamic Patterns in Avian Influenza Viruses

**DOI:** 10.1371/journal.pbio.1001931

**Published:** 2014-08-12

**Authors:** Benjamin Roche, John M. Drake, Justin Brown, David E. Stallknecht, Trevor Bedford, Pejman Rohani

**Affiliations:** 1 Department of Ecology and Evolutionary Biology, University of Michigan, Ann Arbor, Michigan, United States of America; 2 Unité de Modélisation Mathématique et Informatique des Systèmes Complexes (IRD/UMPC 209), Bondy, France; 3 Odum School of Ecology, University of Georgia, Athens, Georgia, United States of America; 4 The Southeastern Cooperative Wildlife Disease Study, Department of Population Health, College of Veterinary Medicine, University of Georgia, Athens, Georgia, United States of America; 5 Vaccine and Infectious Disease Division, Fred Hutchinson Cancer Research Center, Seattle, Washington, United States of America; 6 Center for the Study of Complex Systems, University of Michigan, Ann Arbor, Michigan, United States of America; 7 Fogarty International Center, National Institutes of Health, Bethesda, Maryland, United States of America; Imperial College London, United Kingdom

## Abstract

Avian influenza viruses (AIVs) have been pivotal to the origination of human pandemic strains. Despite their scientific and public health significance, however, there remains much to be understood about the ecology and evolution of AIVs in wild birds, where major pools of genetic diversity are generated and maintained. Here, we present comparative phylodynamic analyses of human and AIVs in North America, demonstrating (i) significantly higher standing genetic diversity and (ii) phylogenetic trees with a weaker signature of immune escape in AIVs than in human viruses. To explain these differences, we performed statistical analyses to quantify the relative contribution of several potential explanations. We found that HA genetic diversity in avian viruses is determined by a combination of factors, predominantly subtype-specific differences in host immune selective pressure and the ecology of transmission (in particular, the durability of subtypes in aquatic environments). Extending this analysis using a computational model demonstrated that virus durability may lead to long-term, indirect chains of transmission that, when coupled with a short host lifespan, can generate and maintain the observed high levels of genetic diversity. Further evidence in support of this novel finding was found by demonstrating an association between subtype-specific environmental durability and predicted phylogenetic signatures: genetic diversity, variation in phylogenetic tree branch lengths, and tree height. The conclusion that environmental transmission plays an important role in the evolutionary biology of avian influenza viruses—a manifestation of the “storage effect”—highlights the potentially unpredictable impact of wildlife reservoirs for future human pandemics and the need for improved understanding of the natural ecology of these viruses.

## Abstract 2

Avian influenza viruses show high genetic diversity, but give rise to pandemic human influenza strains which are much less diverse; we show that these contrasting degrees of viral diversity can be explained by both host (demography) and viral (environmental durability and mutability) factors.

## Author Summary

Human populations have experienced several pandemics involving new subtypes of influenza virus over the past century. All of these pandemic strains contained gene segments that originated in wild birds, a host pool that supports a very large and genetically diverse array of influenza viruses. However, once an avian strain establishes itself within the human population, the genetic diversity of the resulting human subtypes is typically quite low compared to that of their avian counterparts. Here we compare the evolutionary dynamics of human and avian influenza viruses in North America and test different hypotheses that might explain these two contrasting evolutionary patterns. Our analysis shows that a combination of characteristics of the host (especially demography) and virus (such as durability in water and mutability) explains the diversity observed. Using a theoretical model, we show that the combination of the short lifespan of wild birds, and greater durability of viruses in aquatic environments, is key to maintaining the high levels of influenza diversity observed in wild birds.

## Introduction

Seasonal epidemics of influenza viruses are responsible for significant human morbidity and mortality [1]. Owing to their RNA makeup, evolution of influenza A viruses occurs rapidly [2],[3] and is an important driver of their epidemiology [4],[5]. Over the past decade, there has been an extensive effort to understand the concurrent epidemiology and evolutionary trajectory of human influenza viruses [4]–[7], an approach termed “phylodynamics” [8]. Surprisingly, parallel analyses in wild birds, the natural reservoir of influenza viruses [9],[10], are lacking. Such analysis is particularly timely because of the recent recognition of H5N1 and H7N9 avian influenza viruses (AIVs) as pandemic threats [11]–[14].

**Figure 1:**
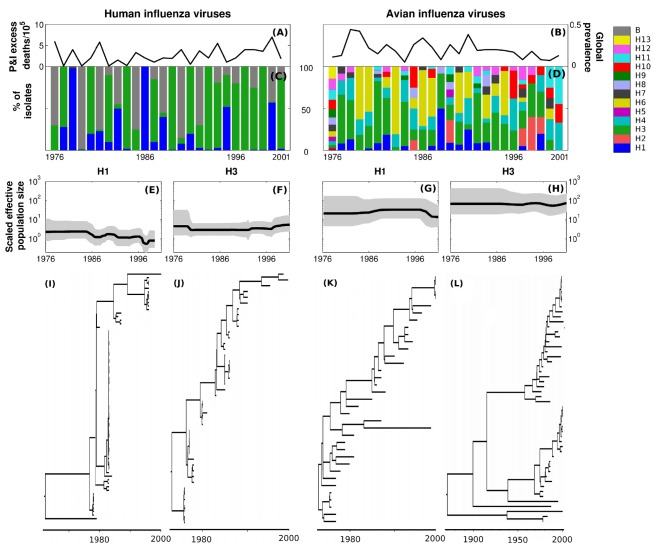
Phylodynamics of human and AIVs sampled within North America between 1976 and 2001 (see Text S1, section S2 for details on dataset). (A and B) Annual variation in North America across all the subtypes for humans [54] and wild birds [55], respectively. (C and D) Dominance patterns of subtypes observed in humans [4] and in birds [55]. (E–H) Scaled effective population sizes scaled by infectious period (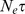), used as a surrogate for antigenic diversity, of H1 and H3 in humans and birds estimated using a nonparametric Bayesian estimator (see Materials and Methods) [58]. (I–L) Corresponding consensus (Maximum Clade Credibility) phylogenetic trees.

The epidemiological and evolutionary histories of human and AIVs in North America from 1976–2001 are summarized in Figure 1. In humans, seasonal influenza outbreaks exhibit substantial annual variation (Figure 1A), which is also reflected in shifting dominance of co-circulating subtypes (Figure 1C). Human influenza viruses exhibit very limited subtype diversity (Figure 1C), as defined by the number of serologically distinct hemagglutinin (H or HA) glycoprotein types [9], where only H1 and H3 subtypes of influenza A viruses have significantly circulated since 1968 [15]. In addition to this paucity of subtypes, genetic diversity is also limited within H1 (Figure 1E) and H3 (Figure 1F) subtypes, as reflected in the slender trunk of the consensus phylogenetic tree (Figure 1I and 1J).

These patterns in human influenza are consistent with “immune escape,” a phenomenon that has been suggested to be common in directly transmitted, immunizing pathogens with a short infectious period, in which antigenic evolution results in partial cross-immunity between strains [8]. In contrast, influenza A viruses in avian populations exhibit a rich array of subtypes, with fully 13 of the known 18 HA subtypes isolated from North American birds over this time span (Figure 1D). This pattern of higher subtype diversity through time is further enriched by higher genetic diversity within subtypes, for instance in H1 (Figure 1G) and H3 (Figure 1H). Indeed, AIVs typically exhibit a scaled effective population size (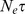, which measures the phylogenetic diversity of the virus population [16]) that is an order of magnitude greater than for their human counterparts: We estimated 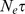 to be 7.0 y and 1.5 y, respectively, for H1 and H3 in humans and 38.7 y and 77.5 y in birds (Figure 1E and 1F; as we show below, other avian subtypes also exhibit higher diversity than commonly observed in H1 and H3 human subtypes). Thus, although phylogenetic trees of H1 and H3 AIVs show some evidence of selection (immune escape) [17], they also document broad viral coexistence (Figure 1K and 1L).

The mechanistic origins of these differences remain unclear. Here, we propose the following nonmutually exclusive hypotheses as possible explanations: (1) The *immunological hypothesis* holds that more rapid loss of immunity and/or weaker heterologous cross-protection in birds than humans reduces competition among strains, leading to higher diversity; (2) the *ecological hypothesis* suggests that associations between virus lineages and avian host species diversity allow contemporaneous evolution within multiple bird species, sustaining an enriched gene pool; (3) the *geographic hypothesis* supposes that greater geographic isolation in birds than in humans leads to allopatric evolution; (4) the *genetic hypothesis* posits that mutation rate differences between avian and human viruses explains the disparity in viral diversity; (5) the *demographic hypothesis* focuses on the higher fecundity and shorter lifespan of birds compared to humans, which may mitigate the selective pressure of herd immunity via substantial recruitment of immunologically naïve individuals propagating the pathogen; and finally, (6) the *epidemiological hypothesis* predicts that there exists a long-lived environmental reservoir for avian strains, but not for human strains, facilitating coexistence of a broad spectrum of genetically, immunologically, and ecologically similar viruses.

To date, there have been no attempts to synthesize the available evidence for or against these different explanations. In this study, we address these hypotheses through a combination of statistical analysis of empirical covariates and epidemiological modeling to identify the most parsimonious explanation for the observed differences of HA genetic diversity between human and AIVs.

## Results and Discussion

### Empirical Covariates of HA Diversity

We analyzed available AIV sequence data from GenBank for 11 HA subtypes from 1976–2013 (see Materials and Methods as well as Text S1, section S2) and developed a set of covariates reflecting specific predictions of the competing hypotheses. We sought to place these covariates on an equal footing and simultaneously to assess the contribution of each to HA diversity using a statistical model. Ordinary linear regression with a large number of covariates (of the same order as the number of observations) results in variance inflation, low statistical power, and issues of statistical identifiability [18]. We therefore adopted a regularized regression method, known as elastic-net regression [19], that solves this problem using a shrinkage estimator to trade off a small amount of bias for substantial reductions in the variance of estimated parameters. As a side effect of this estimation scheme, the resulting coefficients may be interpreted as evidence for or against the inclusion of a covariate (if the coefficient shrinks either to nonzero or zero, respectively). Furthermore, by normalizing covariates we can straightforwardly quantify and compare the relative size of each effect.

This regression analysis yielded the following conclusions (Text S1, sections S4.1–S4.3). As shown in Table 1, among AIV subtypes, each hypothesis we considered made a contribution towards HA genetic diversity, though the magnitude of effects varied considerably (Table S2 for covariates values). Indeed, the strongest covariates were immune selective pressure (Hypothesis 1, quantified via the amino acid substitution rate) and the environmental durability of virions (Hypothesis 6, inferred by experimental incubation data on viral persistence), whose respective impacts were at least twice as big as the effects of nucleotide mutation rate (Hypothesis 4, quantified by multiplication of nucleotide substitution rate by the substitution rate at third position sites), geographic structure (Hypothesis 3, inferred by *F_ST_* that measures population differentiation through space), and host diversity (Hypothesis 2, characterized by Shannon Index of host species sampled).

**Table 1:** Elastic-net multiple regression model testing the association between HA sequence diversity and alternative hypothesized mechanisms.

Hypothesis	Test Variable	Coefficient
Host immunity	Amino acid substitution rate	−0.995
Host diversity	Host Shannon Index	0.111
Geographic structure	Fixation index, (*F_ST_*)	−0.285
Mutation rate	Nucleotide mutation rate	0.276
Transmission ecology	Environmental durability	0.687

The associations tested (see Text S1, section S4 for additional analyses) are between subtype-specific average 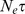 and (I) strength of selection (amino acid subsitution rate), (II) Shannon Index of bird species identified in genetic sequences, (III) geographic population structure (*F_ST_*), (IV) nucleotide mutation rate, and (VI) environmental durability (Rt). All variables have been standardized (converted to a mean of 0 and a variance of 1) to have directly comparable coefficients.

Some of these results are not surprising. For instance, our finding concerning the modest contribution of host species diversity to HA genetic variation had previously been observed by Chen and Holmes [20] and probably arises from frequent interspecies transmission. Similary, the impact of geographic structuring on gene flow among North American AIVs has been elegantly demonstrated elsewhere [20],[21]. The important novel result to emerge from our statistical analyses is the substantial contribution of virus durability to HA genetic diversity. This is a component of virus biology not previously considered in the study of AIV evolution. Thus, to better dissect how the durability of AIVs in the environment affects transmission dynamics and subsequently HA diversity and simultaneously to explore Hypothesis 5 (host demography), we constructed a mechanistic phylodynamic model.

### Computational Model of Influenza Evolution

**Figure 2:**
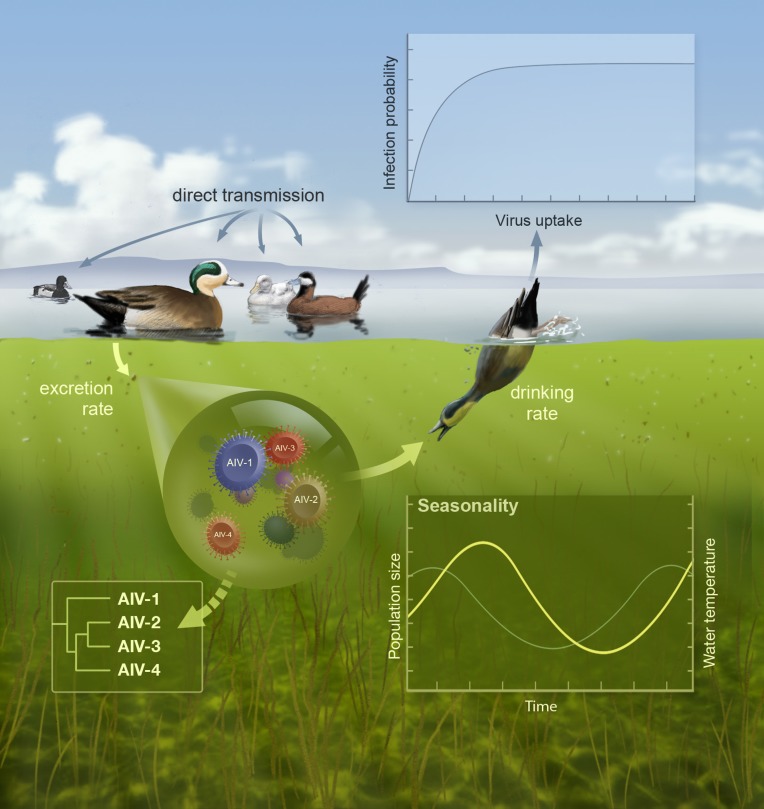
Illustration of our individual-based transmission model. The model takes into account seasonality in host population size (for breeding and migration) and virus durability (driven by fluctuations in temperature), direct (fecal-oral) transmission, and the deposition of virus in the environment, leading to indirect transmission chains with infection probability determined by virus EID_50_. The framework can accommodate multiple host species, but results presented in this study are from a single species model (Text S1, section S3). *Illustration by John Megahan.*

Our model is stochastic, seasonally forced, and agent-based [22] and incorporates a one-dimensional antigenic space, where nonneutral mutations change antigenic phenotype from neighbor to neighbor, thus decreasing cross-immunity (Text S1, section S3) [23]. Crucially, our model allows the tracking of virus antigenic diversity and hence reconstruction of within-subtype digital phylogenies from model output (algorithm detailed in Text S1, section S3.3, Figures S7 and S8), as summarized in Figure 2. Virus diversity is quantified in our model by the number of different antigenic strains at a given time and provides an analog to the diversity inferred by the scaled effective population size on genetic data. This model also enables assessing the role played by host demography (Hypothesis 5) on the maintenance of virus diversity. If these factors are to explain the observed differences between human and avian strains described above, then we expect to observe rapid population turnover and an absence of genetic diversity in a host population parameterized for humans [4],[5],[24], whereas a model parameterized for birds should show broad coexistence of viral strains (parameters are detailed in Table S3). Because the modeling framework we adopt may give rise to either restricted or expansive antigenic diversity depending on epidemiology [23], the inferences one draws are not a result of prejudicial selection of model parameters or functional forms.

An innovative aspect to this model is our formulation of transmission. AIV transmission has been thought to be predominantly fecal-oral, which has been considered as essentially direct because of (i) the proximity between susceptible and infected birds needed for infection and (ii) the scaling of transmission with the duration of infectivity. Furthermore, recent research points to direct bird-to-bird transmission via the respiratory route [25]. Evidence is accumulating, however, to suggest that an additional transmission route is possible via long-lived viruses in environmental reservoirs [26]–[30], effectively giving rise to a second (longer) time scale over which transmission can occur. This hypothesis is based in part on the routine isolation of AIVs from mud samples, soil swabs [26], unconcentrated lake water [31], feathers [32], and the observation of prolonged virus durability in water [9],[33]–[36] and other media [37]. Virus durability is commonly quantified by Rt, which is the time required to reduce infectivity by 90%, and may vary from a couple of days to several months [33]. Rt is determined both by physical environmental conditions, notably temperature, pH, and salinity [33],[34], as well as by subtype identity [38]. Consequently, Hypothesis 6 suggests that environmental transmission could act on a distinctly longer time scale than direct fecal-oral transmission, thereby significantly impacting virus diversity and phylogenetic structure through frequent re-seeding of the avian virus gene pool, as illustrated in Figure S1.

**Figure 3:**
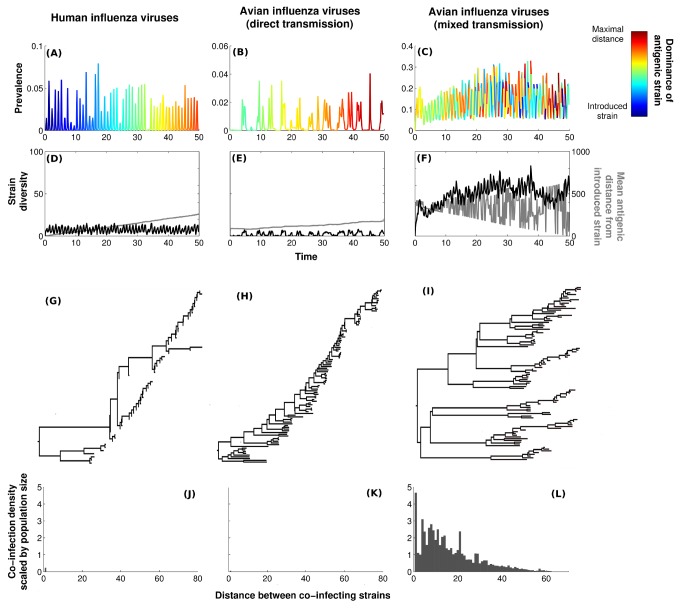
Phylodynamics of our individual-based model (IBM). (A–C) Time series of influenza prevalence in humans, avian system with only direct transmission, and avian system with mixed transmission, respectively. Basic reproduction ratio, *R*_0_, is set to 1.5 for direct transmission, and environmental durability is set at 20 d when this transmission route is included. Colors represent antigenic distance between the introduced strain and the dominant variant at time *t*. (D–F) The black line represents antigenic diversity through time (i.e., number of antigenic strains), whereas the grey line demonstrates temporal changes in the antigenic distance of the dominant strain to the introduced strain. Time is expressed in years. (G–I) Associated reconstructed phylogenies (see Text S1, section S3.3). (J–L) Co-infection patterns for the situations depicted previously. See Materials and Methods for parameter values and Text S1 (section S5) for sensitivity analyses.

To quantify the influence of Hypothesis 5 (host demography) on influenza virus diversity, we first parameterized our model to mimic the within-subtype dynamics of human influenza, assuming only direct transmission. Seeding simulations with only a few antigenic variants, we observed the continual replacement of a dominant strain by new antigenic variants (Figure 3A), driven by selective pressure to escape herd immunity in the host population, as empirically observed [4],[5],[24]. The direct measure of antigenic diversity generated by our model (Figure 3D; six antigenic strains coexist on average) is consistent with our estimates of scaled effective population size of human influenza (Figure 1E or 1F). The resulting inferred phylogenetic tree from our model output (Figure 3G) is also “ladder-like,” characteristic of the strong immune escape signature observed in data (Figure 1I or 1J).

We then addressed demographic explanations by exploring the impact of host biology alone, reparameterizing the model to take into account the reduced lifespan, increased fecundity, and seasonal breeding of birds compared with humans. Model output remained qualitatively unaffected, demonstrating continuous antigenic evolution (Figure 3B), with low-standing antigenic diversity (Figure 3E; five strains coexist on average) and a slender trunk in the phylogenetic tree (Figure 3H). Thus, in this model, host demographic properties alone do not strongly influence levels of genetic variation. In contrast, we found that the inclusion of environmental transmission dramatically increased standing antigenic diversity of AIVs (Figure 3C and 3F; 60 strains coexist on average), resulting in both immune selection and virus diversification (Figure 3I). The phylogenetic tree contains lineages that would have gone extinct in the absence of environmental transmission, demonstrating the punctuation of antigenic evolution with reintroduction of past dominant variants, to which there is little immunity in the population (Figure S1).

**Figure 4:**
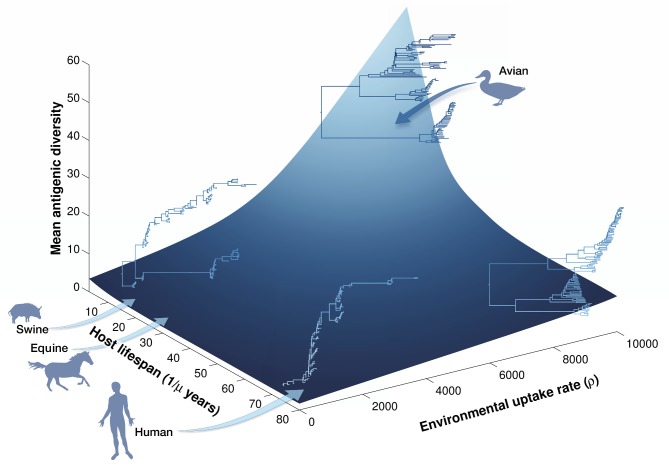
Sensitivity of simulated pathogen diversity to host lifespan and environmental transmission. Simulations were run for 50 y, with the first 10 y discarded as transients. We assumed a population size of 10^4^ individuals, with no seasonality in demography or environmental durability. The *z*-axis corresponds to the mean antigenic diversity over the 40-y period. A decrease in environmental uptake rate (*x*-axis) dramatically reduces mean antigenic diversity (average of the number of strains) and switches the phylogenetic pattern from one that depicts broad coexistence to an immune escape profile. This switch in phylogenetic pattern is most pronounced when host lifespan (*y*-axis) is short. To estimate digital phylogenies, 100 strains were sampled over the last 25 y of simulations. Other parameters are as in Figure 3. Surface has been drawn through a 3D cubic interpolation.

Next, we carried out sensitivity analyses spanning the parameter space of the most common influenza systems, including swine and equine influenza (Figure 4). We found that the long natural lifespan (and low fecundity) of free living mammalian hosts sustain the selective pressure exerted by herd immunity in the population on dominant strains. Indeed, in the presence of long lifespan and associated long-lived immunity, even substantial levels of environmental transmission do not dramatically increase antigenic diversity. Reduced host lifespan, however, leads to a faster turnover of the population, reducing the selective impacts of herd immunity. As shown in Figure 4, it is the combination of high lifetime fecundity and environmental transmission that produces dramatic increases in genetic diversity and the coexistence of distantly related viral lineages. In Text S1 (section S5, Figures S11, S12, S13, S14, S15, Table S4), we present results of sensitivity analyses to demonstrate the robustness of this broad conclusion to changes in assumed duration of immunity, the strength of cross-protection, the infectious period, direct transmission rate, and the mutation rate. This result also shows very little variation in stochastic realizations of the model.

### Phylogenetic Signatures of Environmental Transmission

**Figure 5:**
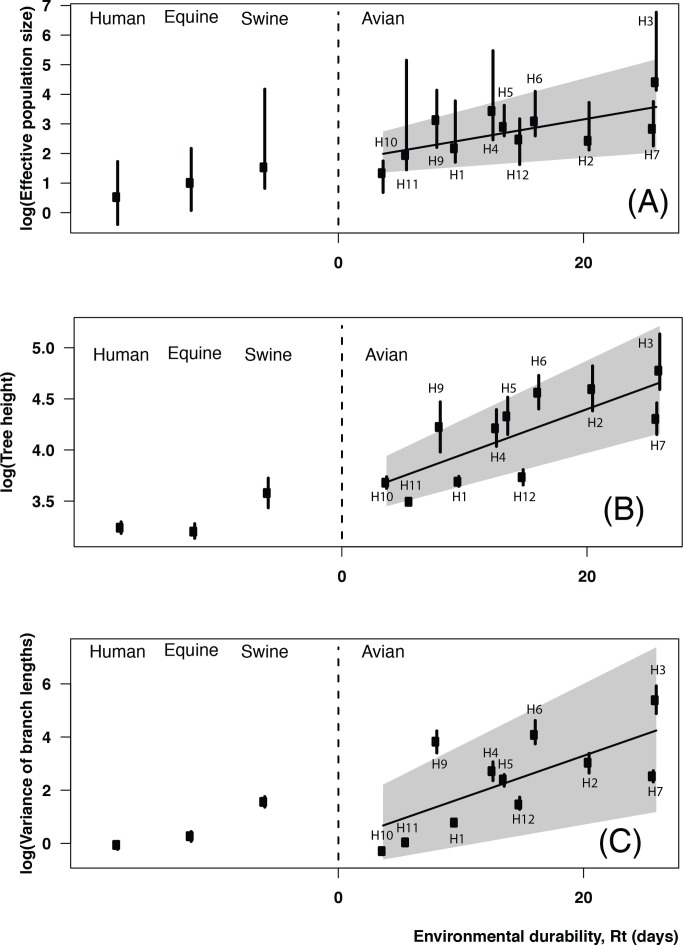
Magnitudes of environmental durability of influenza subtypes and their consequences for evolutionary patterns observed in sequence data. (A) Under natural physical conditions (temperature, 20°C; salinity, 0; pH 7.2, no seasonality), increased environmental durability (captured by Rt) is associated with higher scaled effective population size (linear relationship with a coefficient of 0.07 and a correlation coefficient of *r* = 0.65, *p* = 0.029). As shown in S5.4, assuming a seasonally weighted value of Rt does not change this association. (B) Environmental durability is correlated with tree height (the sum of branch lengths from the tip to the root), which indicates an increase in antigenic diversity (linear regression with a slope at 0.043, correlation coefficient of *r* = 0.75, *p* = 6.83×10^−3^). (C) The variance in branch lengths increases with virus durability, indicating variability in transmission time scales (linear regression with a coefficient of 0.16 and correlation coefficient of *r* = 0.68, *p* = 0.019). Confidence intervals show the 75% quantile in every panel. HAs for humans (H3), equines (H3), and swines (H1) are the most prevalent Hemagglutinin subtypes for these hosts.

Four testable predictions arise from our model (Figure 4), three of which can be explored using our existing data set. First, viruses with greater environmental durability are predicted to exhibit greater genetic diversity when host lifespan is short (Figure 5A). Second, increasing viral durability in the environment is predicted to facilitate the reintroduction of past virus variants and hence to correlate with the estimated time to the most recent common ancestor (TMRCA). Third, the variable nature of indirect transmission chains via the environmental reservoir [39],[40] is predicted to generate greater variability in branch lengths. Fourth, and finally, environmental transmission should increase the frequency of co-infection events between antigenically distant viruses (Figure 3J–L) and therefore the number of co-circulating subtype combinations.

We tested for the presence of the first association using phylogenies inferred for 10 different avian influenza HA subtypes isolated from North American wild birds (Text S1, section S2). For comparison, we performed a parallel analysis of the most prevalent subtype observed in human (H3), equine (H3), and swine (H1) influenza viruses. Recalling that HA durability correlates with HA genetic diversity (Table 1 and Figure S10), we further observed that scaled effective population size (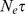) increases with HA durability either estimated at a fixed temperature (Figure 5A) or averaged over a season (Figure S16 and Table S15). Here, *N_e_* represents the size of an idealized population corresponding to observed levels of genetic diversity, and τ represents the generation time of the virus. Because long-term transmission chains resulting from environmental durability should directly increase AIV generation time (the average time between infections), a correlation between 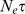 and experimentally measured environmental durability is evidence for the environmental transmission hypothesis. An increase in 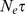 is expected to impact the time it takes for the sampled viruses to coalesce to a common ancestor. Consistent with the second model prediction, we observed that TMRCA, quantified by tree height here, increased with HA durability (Figure 5B). Third, we found that variance in branch lengths correlated with HA durability across subtypes (Figure 5C), as predicted.

One final model prediction, that co-infection frequency should increase with environmental durability, is of great evolutionary relevance, as co-infection is necessary for reassortment, which may be a prerequisite to the evolution of pandemic strains [17],[41]. Although co-infections are expected to occur infrequently and mainly between related strains in humans (Figure 3J), environmental transmission in avian communities allows for substantially more frequent co-infections, especially between antigenically distant variants (Figure 3L). This is due to effects of environmental transmission increasing the propensity for co-infection (an empirically supported phenomenon [42]), together with the high antigenic diversity generated by environmental transmission (Figure 4). To test this prediction, a regression of subtype-specific HA diversity against the entropy of associated NA subtypes failed to detect any significant relationship (*r* = 0.15, *p* = 0.65).

### Conclusions

Our study identified the mechanisms that act to determine hemagglutinin genetic diversity in avian influenza viruses. In particular, the analyses reveal that our hypotheses act in concert to shape the phylodynamics of AIVs. These results are consistent with prior studies that have examined each mechanism in isolation. For instance, it has been shown that strong spatial structuring is an important factor in the phylogeography of these viruses [20],[21]. Similarly, the modest association between genetic diversity and host species number is known [20]. Although it is widely hypothesized that increasing antigenic evolution decreases genetic diversity across human subtypes [43] and between human and swine H3 [44], our research has also demonstrated that increasing avian immune selective pressure acts to reduce influenza virus diversity.

Our most surprising empirical finding is that HA genetic diversity increases with virus durability, as measured in experimental assays, across AIV subtypes (Table 1 and Figure 5). The corresponding theoretical result is that, in short-lived hosts, increasing the frequency of environmental transmission results in greater equilibrium levels of viral genetic diversity (Figures 3 and 4). Thus, both empirical and theoretical results suggest that environmental transmission acts in wild bird populations to increase avian influenza genetic diversity. As emphasized throughout, our results do not exclude a role for additional mechanisms (e.g., Hypotheses 1–3), but establish statistically that the size of the effects of subtype-specific amino acid substitution rates and environmental durability are largest.

Two limitations of this study warrant further investigation. First, a detailed understanding of cross-immunity remains an important empirical limitation to any study of avian influenza evolution. Establishing the duration and extent of protective immunity against heterotypic viruses, particularly, remains a priority. At present, comparative data to test this idea directly are lacking, although work showing the absence of immunity conferred by DNA subtype-specific vaccines to challenge strains from other subtypes [45] suggests that the effect of cross-immunity will be limited. Second, our model assumed a one-dimensional strain space for the practical purpose of numerical tractability [23]. Future work should investigate the effects of incorporating other structures of cross-immunity [4],[5] into epidemiological models of across-species influenza. Similarly, our findings show the relative importance of environmental persistence on phylodynamics of AIVs. Crucially, viral persistence may also occur in nonaquatic environments, including lake sediment, feathers, and feces [26],[32],[37].

Elsewhere, it has been shown that the environmental reservoir can be a crucial source for sparking off annual outbreaks [39] and may have an impact on interannual AIV durability [46]. As we have demonstrated here, another consequence of this feature of AIV transmission is broad strain coexistence that is similar to an ecological phenomenon called the “storage effect” [47],[48]. This effect has been identified for soil bacteria, where dormancy is thought to generate a high level of microbial diversity [49]. Dramatically, one of the main predictions of the storage effect theory is that large fluctuations in recruitment rates are expected of low-density species [48]. Indeed, as shown in Figure 3C, at any given time, the dominant strain may have first appeared far in the past, and as shown in Figure S9, this pattern of dominance is not predictable. Within an epidemiological context, this suggests that unpredictable outbreaks of rare subtypes may occur due to the absence of herd immunity.

Finally, our findings have practical implications for the management of influenza in wild birds. Particularly, our results indicate that—in addition to movement restrictions [50] and measures aimed at population size [51]—considering an environmental dimension to AIV control may be advisable [31]. Particularly, contaminated environments may remain infectious for an extended period following the cessation of transmission among hosts. If complete elimination of the virus is desired, then environmental decontamination may be required. Because of the outer lipid envelope associated with influenza viruses, chlorination has been proposed as a potentially effective method for decontamination [52],[53]. Given the impossibility of large-scale field trials, simulation exercises using models such as we report here may be crucial for determining whether such methods are indeed practically feasible.

## Materials and Methods

### Epidemiological Time Series

The human epidemiological time series presented in Figure 1 is the death rates attributed to pneumonia and influenza reported in the United States [54]. This measure is known to correlate with human influenza activity [54], enabling qualitative description of population-wide influenza transmission. The subtype dominance patterns focus also on the United States and have been estimated through an annual sampling performed by the Center for Disease Control and Prevention [4]. Avian epidemiological time series and subtype dominance has focused on a duck population sampled in Alberta, Canada [55].

### Phylogenetic Analyses

For the phylogenetic analyses presented in Figure 1, we focused on the HA gene for 10 subtypes sampled from wild bird species in North America between 1976 and 2001. We restricted our attention to this period because of the availability of parallel subtype-specific AIV prevalence data. For the remaining phylogenetic analyses (such as those presented in Table 1 and Figure 5), we examined sequence data from 1976–2013 from North America (United States and Canada) for avian, swine, and equine subtypes (Figures S2, S3, S4, S5, S6 and Table S1). For human influenza viruses, we considered only sequences from Memphis, Tennessee, in order to use a comparable number of sequences. Data for avian subtypes are available from the Dryad Digital Repository (http://dx.doi.org/10.5061/dryad.dryad.8ct18 [56]). Phylogenetic trees have been computed using the software BEAST [57] assuming a strict molecular clock [58], a site heterogeneity that is gamma distributed, a HKY substitution model, and a Bayesian Skyline Plot (BSP) with 10 groups [59]. The number of replicates was adjusted to maximize effective sample size; 5 millions replicates were used for burn-in.

### Estimation of Adaptive Evolution

We measured nucleotide substitution rate at third position sites as a proxy for nucleotide mutation, as evolution at third position sites should primarily represent synonymous change [60]. We also measured amino acid substitution rate, which is affected by both nucleotide mutation rate and selective effect of mutations. In the regression analysis, we find that amino acid substitution rate has a much stronger correlation with 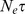 than does third position nucleotide substitution rate (coefficients −0.99 versus 0.27; Table 1). This suggests that it is the differing levels of selection on different HAs that determines viral diversity, rather than differing intrinsic mutation rates. The finding of a strong negative correlation between amino acid substitution rate and 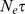 is consistent with the action of position selection driving amino acid replacements and purging diversity from viral populations [16].

### Experimental Protocol for Determining Environmental Durability

Data on environmental durability for each subtype come from experimental data [38]. For each virus subtype, infective virions were diluted 1∶100 in water samples. The inoculated water samples were then divided into 3.0 ml aliquots in 5.0 ml polystyrene tubes and placed in incubators set to the appropriate treatment temperature. For each virus temperature trial, the viral inoculated water was sampled at the time of viral inoculation and at a second time point postinoculation. Titrations at all time points were performed in duplicate. The second postinoculation time point varied with each trial and was determined based on prior estimates of the time required for the titer of the virus in the water sample to be reduced by at least 1 log10 TCID50/ml [33],[35]. Duplicate 0.5 ml samples of AIV-inoculated water were diluted 1∶1 by addition of 0.5 ml of 2× serum-free MEM. Ten-fold dilutions (10^−1^ to 10^−8^) were then made in 1× MEM supplemented with antibiotics. These titers were used to estimate Rt as the time required to reduce infectivity by 90%, assuming a linear association.

### Regularized Regression Analyses

The goal of regression analysis was to estimate the size of statistical effect on AIV diversity of covariates corresponding to alternative causal hypotheses. As described above, a covariate corresponding to each hypothesis was developed and assessed for each antigenic subtype. These covariates were (i) *F_ST_* (the proportion of genetic variance contained in a subpopulation relative to the total variance) of each subtype (geographic hypothesis), (ii) number of species where strains have been sampled (host diversity hypothesis), (iii) subtype-specific nucleotide mutation rate (genetic hypothesis), (iv) amino acid substitution rates characterizing an immune selective pressure (immunological hypothesis), and (v) environmental durability Rt under natural physical conditions (temperature, 20°C; salinity, 0; pH 7.2; epidemiological hypothesis). Due to correlations among these variables, univariate analysis was not considered to provide reliable estimates of covariate effects. But ordinary least squares multiple regression would be equally ill-advised, resulting in weakly identifiable parameters and variance inflation. A generic solution to this problem is provided by penalized least squares models, such as ridge regression and elastic-net regression. These methods introduce a new estimator, which differs from the maximum likelihood estimator by an additional penalty. In effect, the penalized estimator trades a small amount of bias for a large reduction in the variance of the estimated coefficients. We chose to use elastic-net regression, which takes the maximum likelihood and ridge regression estimators as limit cases and therefore can be fine-tuned to balance the bias-variance tradeoff. Fitting of an elastic-net regression model requires the estimation of an additional tuning parameter (the penalty coefficient), which was numerically selected using cross-validation following [19]. The outcome of this procedure is a statistical model with coefficients shrunk to minimize generalization error. Covariates for which shrunk coefficients are zero can be inferred to have no effect.

### Individual-Based Modeling and Digital Phylogenies

The individual-based model developed here has been shown to generate evolutionary dynamics that are not statistically distinguishable from the classic *SIR* model in the limiting cases where analogous mathematical models can be still formulated [22]. Its main algorithm is detailed in Text S1 (section S3.1). Only nonneutral antigenic mutations have been explicitly considered within the model. The reconstruction of neutral mutations to infer the digital phylogenies has been implemented in a second step, detailed in Text S1, section S3.3. To avoid definitive extinctions, immigration of infectious individuals was included, with immigrant strains randomly selected according to the proportion of each variant present during the previous epidemic in order to avoid a strong influence of infectious immigration. Simulations start with four different strains far enough to avoid cross-immunity between them (see Text S1, section S3.2).

### Parameter Values of Model

For human settings, we have assumed a constant population size of 10^6^ individuals, with a mean lifespan of 80 y and a transmission rate of *β*(*t*) = 7.8.10^5^(1+0.035cos(*t*)) [5]. The avian community is assumed to contain 10^4^ individuals (host lifespan is 4 years) [39] with a seasonal demography integrated through a fluctuating birth rate *b*(*t*) = *b*(1+0.8·sin(*t*)) [30]. In both cases, β(*t*) has been chosen to ensure 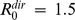 on average, as reported from previous studies [4],[30],[39]. Environmental transmission is characterized by an uptake rate of 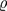/*L* = 6.73 [30] and an environmental durability ξ(*t*) = 20×(1+0.9·sin(*t*)) (20 d on average [38]). To infer digital phylogenies, 100 strains have been sampled over the last 25 y from simulation runs.

## Supporting Information

Figure S1 Conceptual summary of study findings. The figure depicts the contrasting transmission dynamics of human (top panels) and avian (bottom panels) influenza viruses. When host lifespan is long and transmission is only via direct contact (as is the case with human influenza viruses), herd immunity to a given antigenic variant produces strong selection pressure for immune evasion, as indicated by strain replacement events in the top panel. With AIVs, however, the long-term environmental reservoir leads to the episodic introduction of older lineages and facilitates viral coexistence.
(TIF)

Figure S2 Temporal distribution of all sampled sequences.
(TIF)

Figure S3 Spatial distribution of avian influenza isolates.The areas shaded in red indicate that this state/province has been sampled.
(TIF)

Figure S4 Summary of environmental durability dataset (from [38]).
(TIF)

Figure S5 Maximal likelihood trees for AIVs ranked from H1 to H6. These trees have been calculated with PhyML.
(TIF)

Figure S6 Phylogenetic trees for AIVs ranked from H7 to H12 (note: H8 has been excluded due to paucity of sequences). These trees have been calculated with PhyML.
(TIF)

Figure S7 Illustration of neutral mutation reconstruction for inference of digital phylogenies.
(TIF)

Figure S8 Test of neutral mutation reconstruction algorithm. Panels on the left represent model parameters leading to a perfect immune escape pattern (each strain is replaced at the next time step). Panels on the right depict viral coexistence. The phylogenies reconstructed (Bottom) through the algorithm detailed in Figure S7 are consistent with the evolutionary dynamics considered (Top). Ten strains are sampled every year.
(TIF)

Figure S9 Antigenic dynamics of the three configurations studied and represented in Figure 3 by digital phylogenies as well as an intermediate situation with a shorter environmental durability at 12 d.
(TIF)

Figure S10 Influence of shrinkage on coefficient values. (Left) Mean-squarred error according the log(λ). Optimal value is for log(λ) = −6.88. (Right) Coefficient values for different levels of log(λ); shaded area represents coefficients for the optimal value of log(λ).
(TIF)

Figure S11 Phylodynamics simulated with different hypotheses than environmental transmission. Parameters are the same as in Figure 3 (A) without environmental transmission. (B) A temporary immunity of 6 mo is assumed. (C) Mutation rate is set at 2.10^−5^ per base per day on 12,000 bases (assuming the whole genome is coding for antigenic variation) instead of 2,000 (assuming that only mutation on HA is coding). (D) Less efficient cross-protection (changing the cross-immunity parameter *d* = 1, see eqn. S1 in Text S1). (E) Longer infectious period (20 d) by keeping R0 of direct transmission constant. (F) With environmental transmission. Co-infection patterns are depicted by the absolute numbers of co-infection events in order to complete the figures shown in the main text, where number of co-infections are scaled by host population size.
(TIF)

Figure S12 Phylodynamics simulated with environmental transmission and different values for key parameters. Parameters are the same as in Figure 3 with environmental transmission. (B) Infectious period is set at 3 d. (C) Infectious period is set at 7 d. (D) Parameter θ (see eqn S1 in Text S1) is set at 0.5 instead of 0.7. (E) Parameter *d* (see eqn S1 in Text S1) is set at 5 instead of 3. Co-infection patterns are depicted by the absolute number of co-infection events in order to complete the figures shown in the main text, where number of co-infections are scaled by host population size.
(TIF)

Figure S13 Comparable simulation to Figure 3F, but with the lower values of mutation rate, as assumed in [5].
(TIF)

Figure S14 Same simulation as for Figure 3A,D, but with 5 million individuals instead of 1 million.
(TIF)

Figure S15Phylodynamics pattern generated for avian configuration (A) without and (B) with environmental transmission. (C) Considering direct transmission with an increased transmission rate (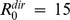) does not generate a notable increase in strain diversity.(TIF)

Figure S16 Environmental durabilities estimated when temperature fluctuates through a cosinus function (with an average of 20 degrees) with an amplitude of 0 (black lines), 0.2 (blue lines), and 0.5 (red lines).
(TIF)

Table S1 Number of sequences for each avian subtype.
(XLSX)

Table S2 Parameters of the model. Values displayed here are used throughout the manuscript, except when sensitivity of parameter is explored. NA, not applicable.
(XLSX)

Table S3 HA subtype and corresponding statistics.
(XLSX)

Table S4 Medium, minimal, and maximal values of strain diversity for the different extreme configurations across 10 replicates. “Human demography” assumes a lifespan of 80 y, and “Avian demography” assumes a lifespan of 4 y. Strong environmental transmission assumes a drking rate of 10^4^ centileters per day.
(XLSX)

Table S5 Results of the multiple regression analysis with a different amplitude of temperature seasonality.
(XLSX)

Text S1 Model description and validation, and supplementary statistical and theoretical analyses.
(PDF)

## Abbreviations

AIVs: avian influenza viruses
HA: hemagglutinin
NA: neuraminidase
