# Supplementary figures and images for: Adaptive Evolution and Environmental Durability Jointly Structure Phylodynamic Patterns in Avian Influenza Viruses
Source: PLoS Biol. 2014 Aug 12;12(8):e1001931. doi: 10.1371/journal.pbio.1001931 (PMC4130664; doi:10.1371/journal.pbio.1001931)

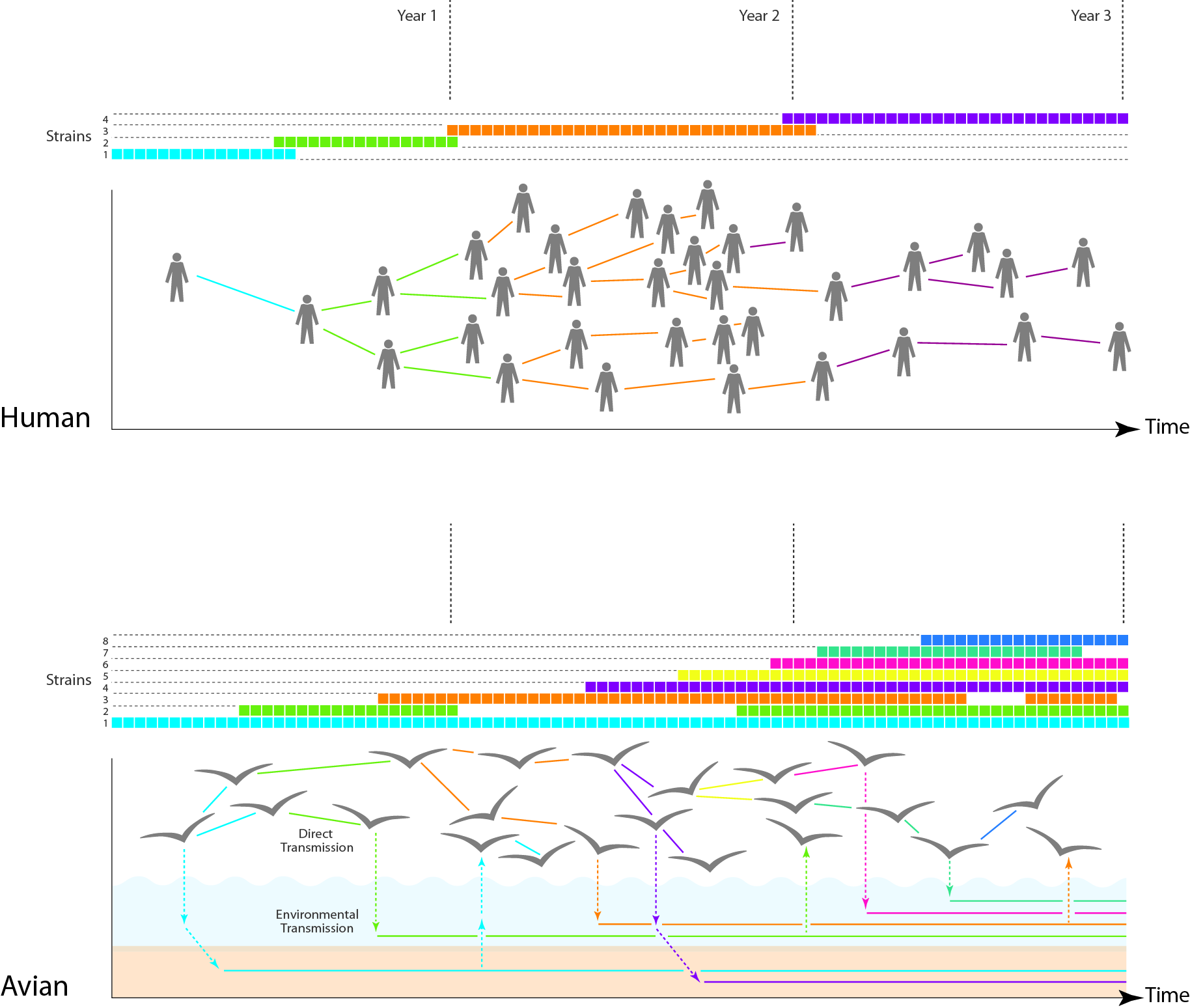

Supplement: Figure S1 — Conceptual summary of study findings. The figure depicts the contrasting transmission dynamics of human (top panels) and avian (bottom panels) influenza viruses. When host lifespan is long and transmission is only via direct contact (as is the case with human influenza viruses), herd immunity to a given antigenic variant produces strong selection pressure for immune evasion, as indicated by strain replacement events in the top panel. With AIVs, however, the long-term environmental reservoir leads to the episodic introduction of older lineages and facilitates viral coexistence. (TIF) [file pbio.1001931.s001.tif]

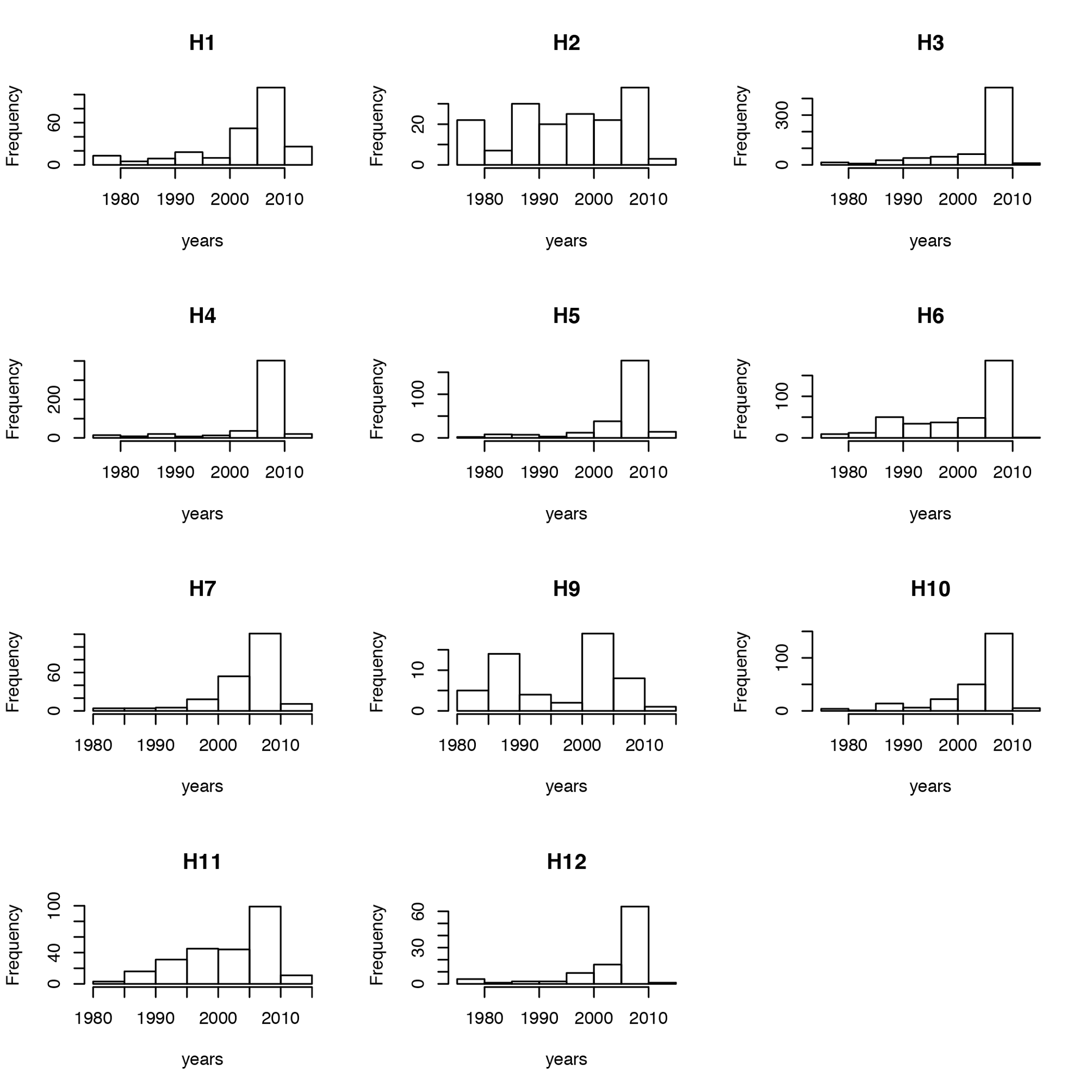

Supplement: Figure S2 — Temporal distribution of all sampled sequences. (TIF) [file pbio.1001931.s002.tif]

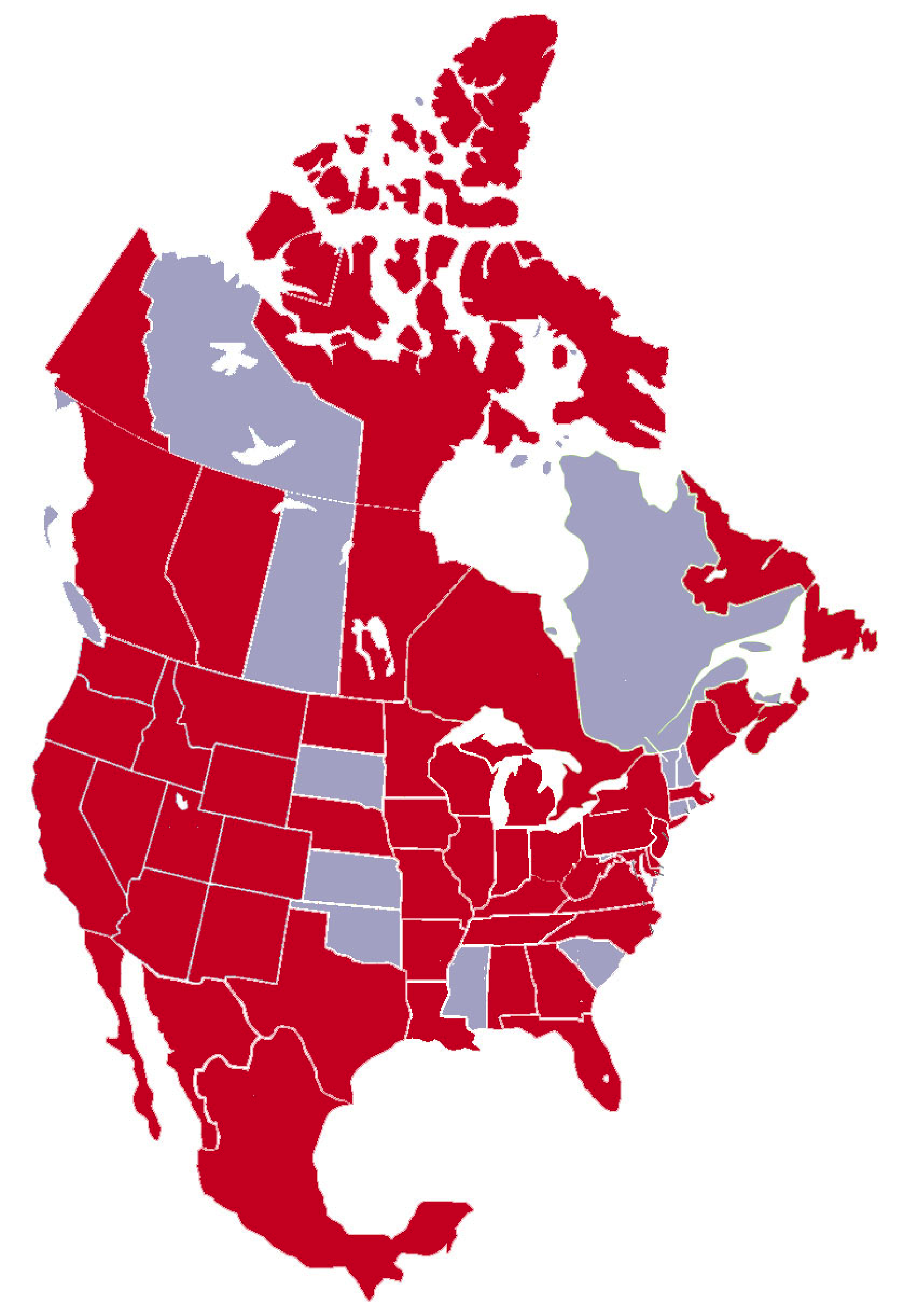

Supplement: Figure S3 — Spatial distribution of avian influenza isolates.The areas shaded in red indicate that this state/province has been sampled. (TIF) [file pbio.1001931.s003.tif]

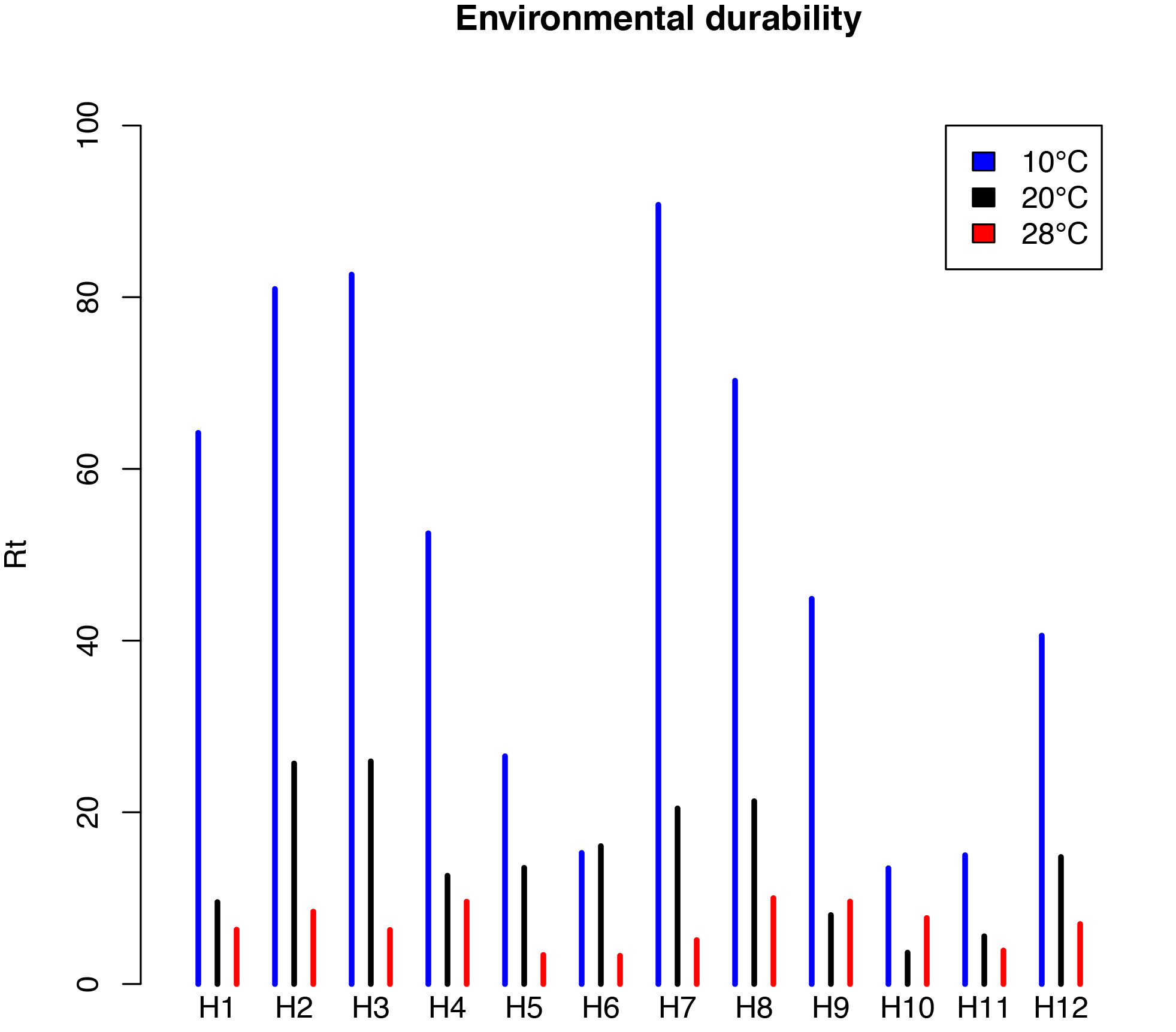

Supplement: Figure S4 — Summary of environmental durability dataset (from [38]). (TIF) [file pbio.1001931.s004.tif]

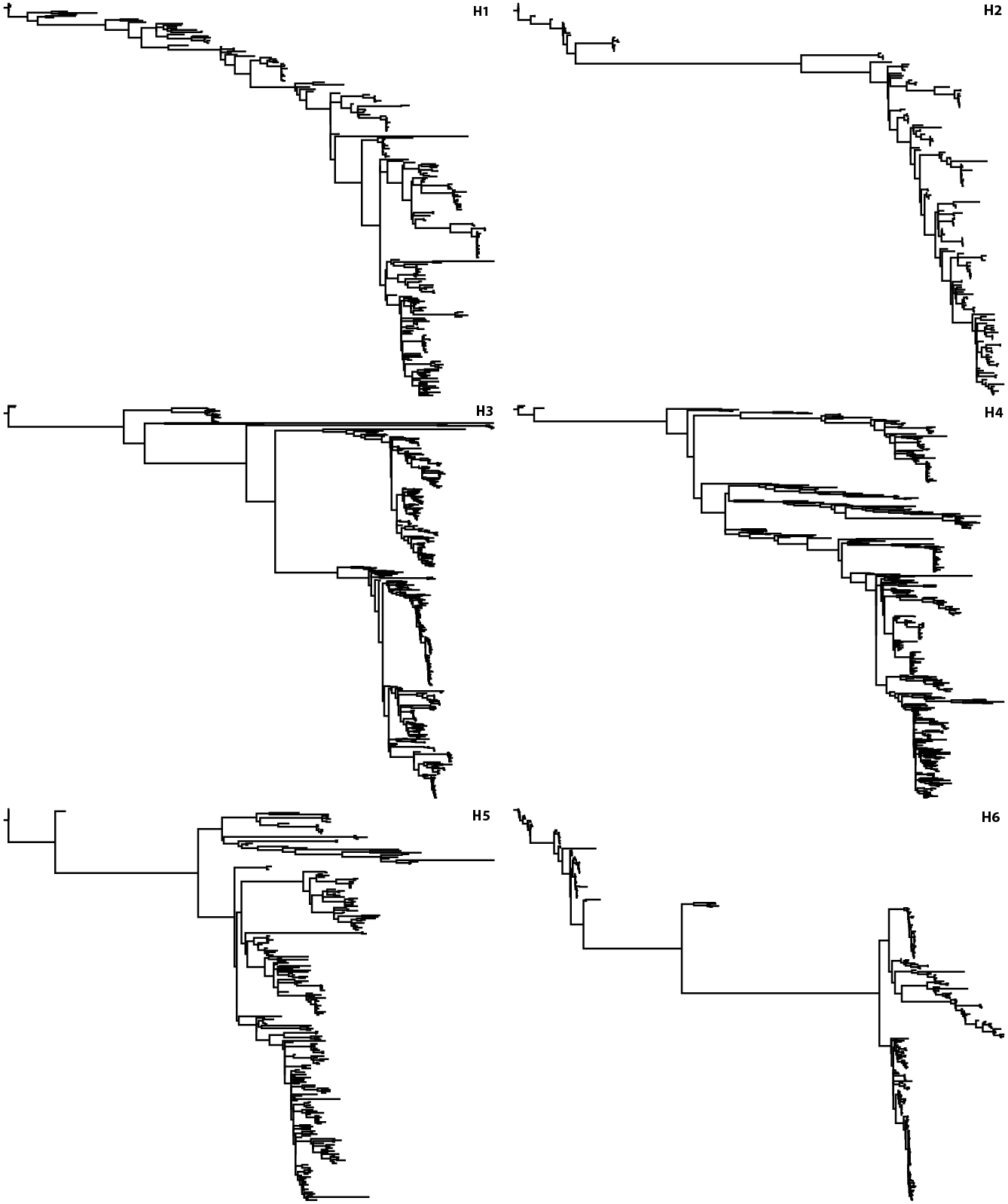

Supplement: Figure S5 — Maximal likelihood trees for AIVs ranked from H1 to H6. These trees have been calculated with PhyML. (TIF) [file pbio.1001931.s005.tif]

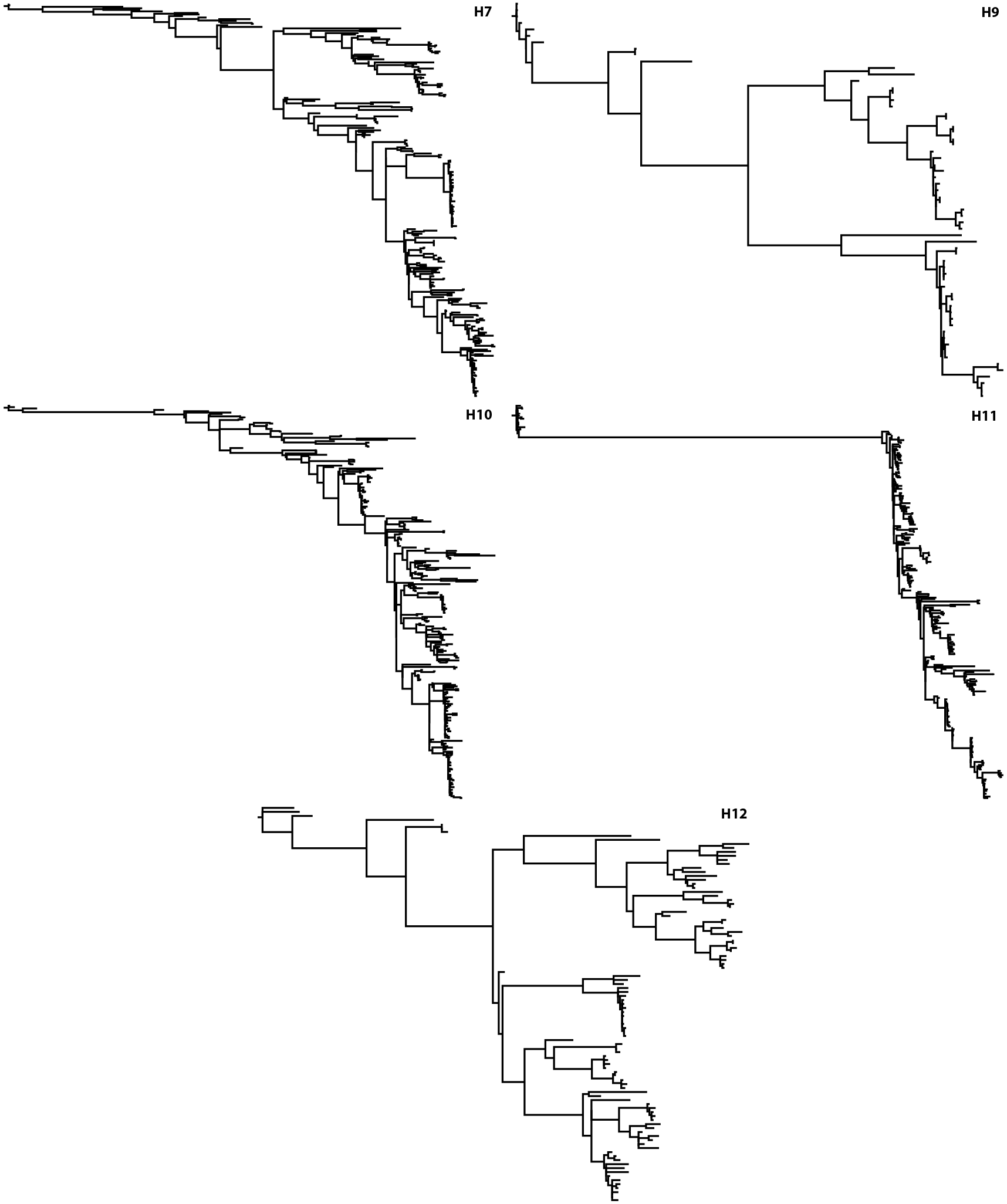

Supplement: Figure S6 — Phylogenetic trees for AIVs ranked from H7 to H12 (note: H8 has been excluded due to paucity of sequences). These trees have been calculated with PhyML. (TIF) [file pbio.1001931.s006.tif]

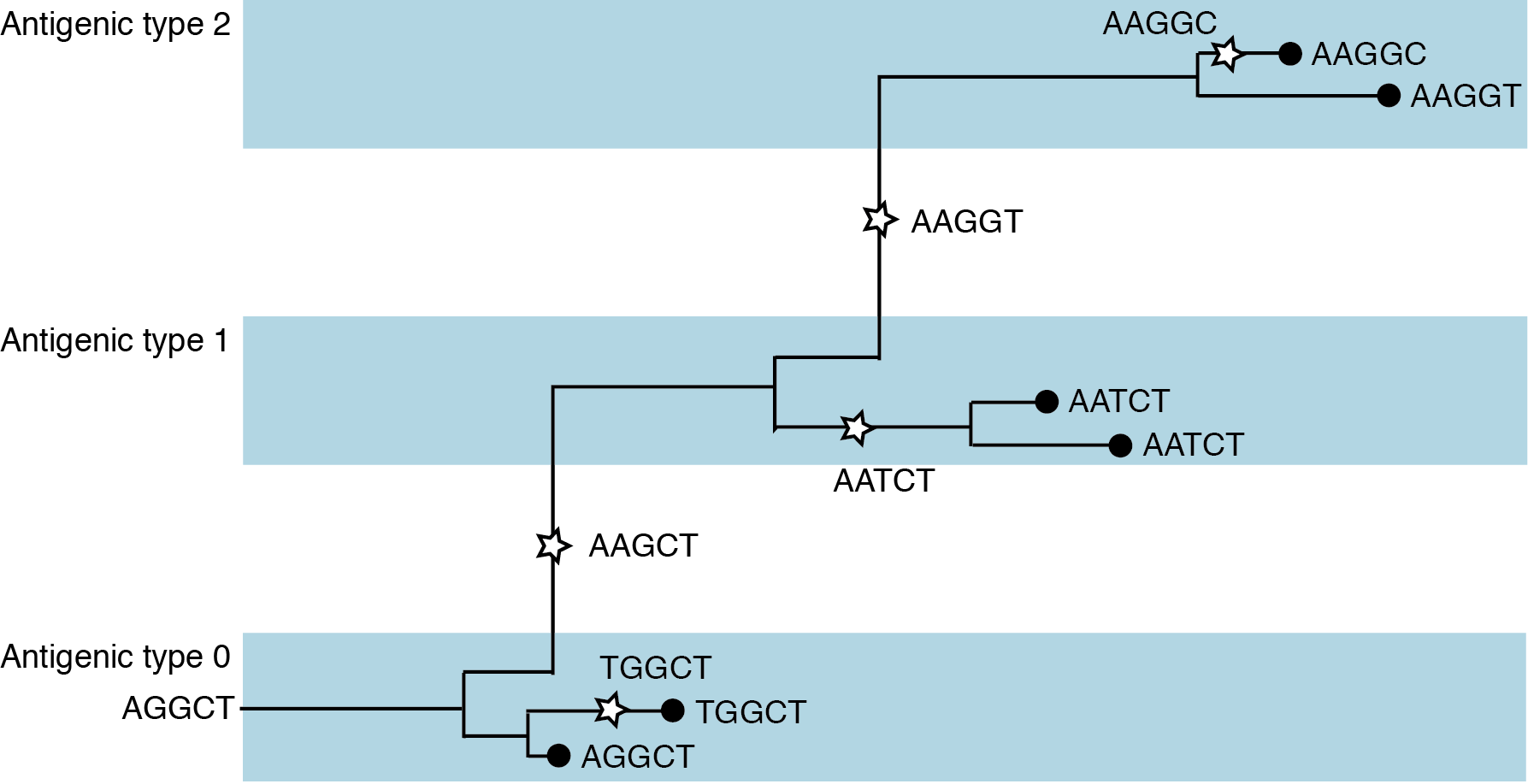

Supplement: Figure S7 — Illustration of neutral mutation reconstruction for inference of digital phylogenies. (TIF) [file pbio.1001931.s007.tif]

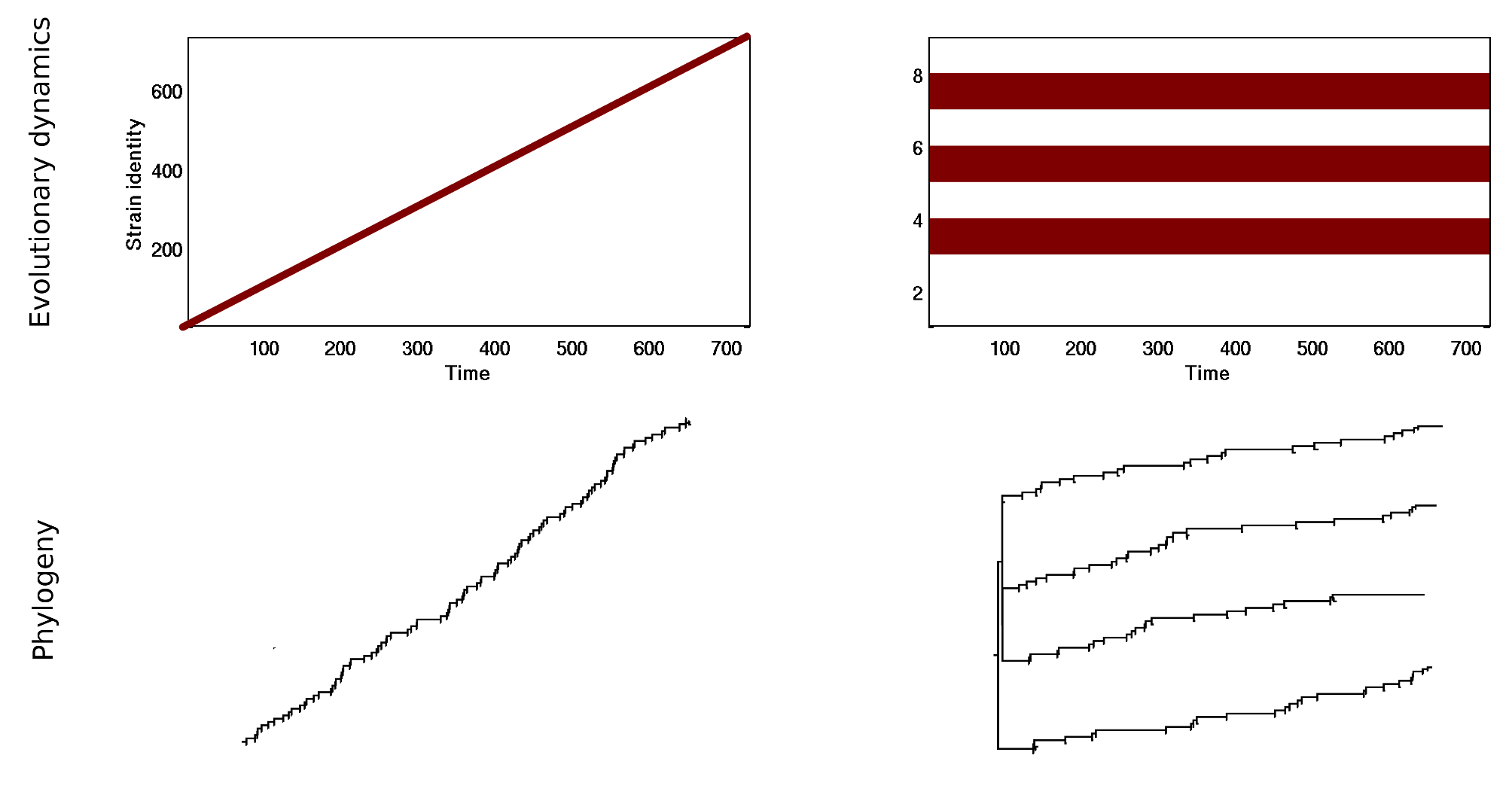

Supplement: Figure S8 — Test of neutral mutation reconstruction algorithm. Panels on the left represent model parameters leading to a perfect immune escape pattern (each strain is replaced at the next time step). Panels on the right depict viral coexistence. The phylogenies reconstructed (Bottom) through the algorithm detailed in Figure S7 are consistent with the evolutionary dynamics considered (Top). Ten strains are sampled every year. (TIF) [file pbio.1001931.s008.tif]

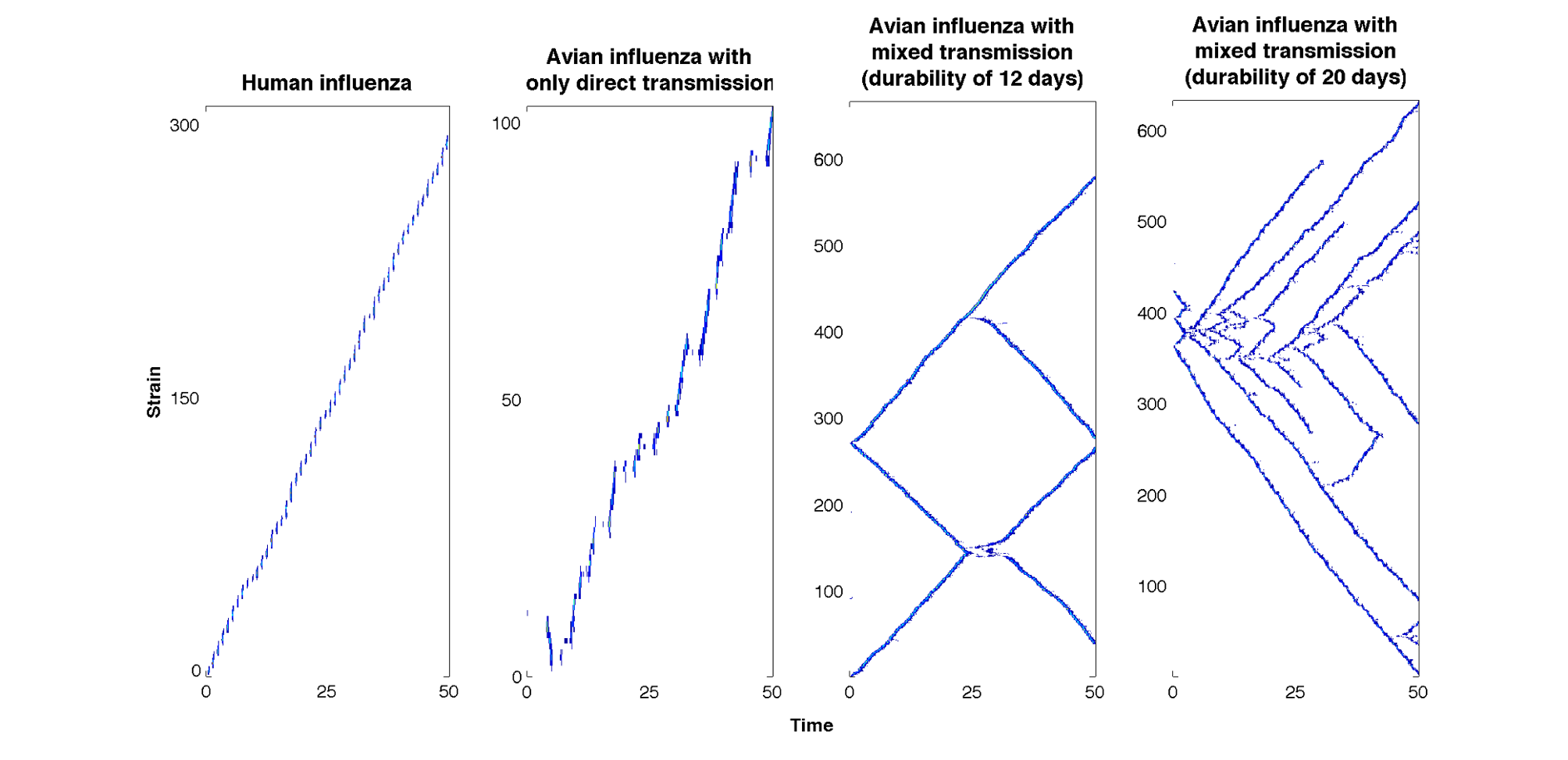

Supplement: Figure S9 — Antigenic dynamics of the three configurations studied and represented in Figure 3 by digital phylogenies as well as an intermediate situation with a shorter environmental durability at 12 d. (TIF) [file pbio.1001931.s009.tif]

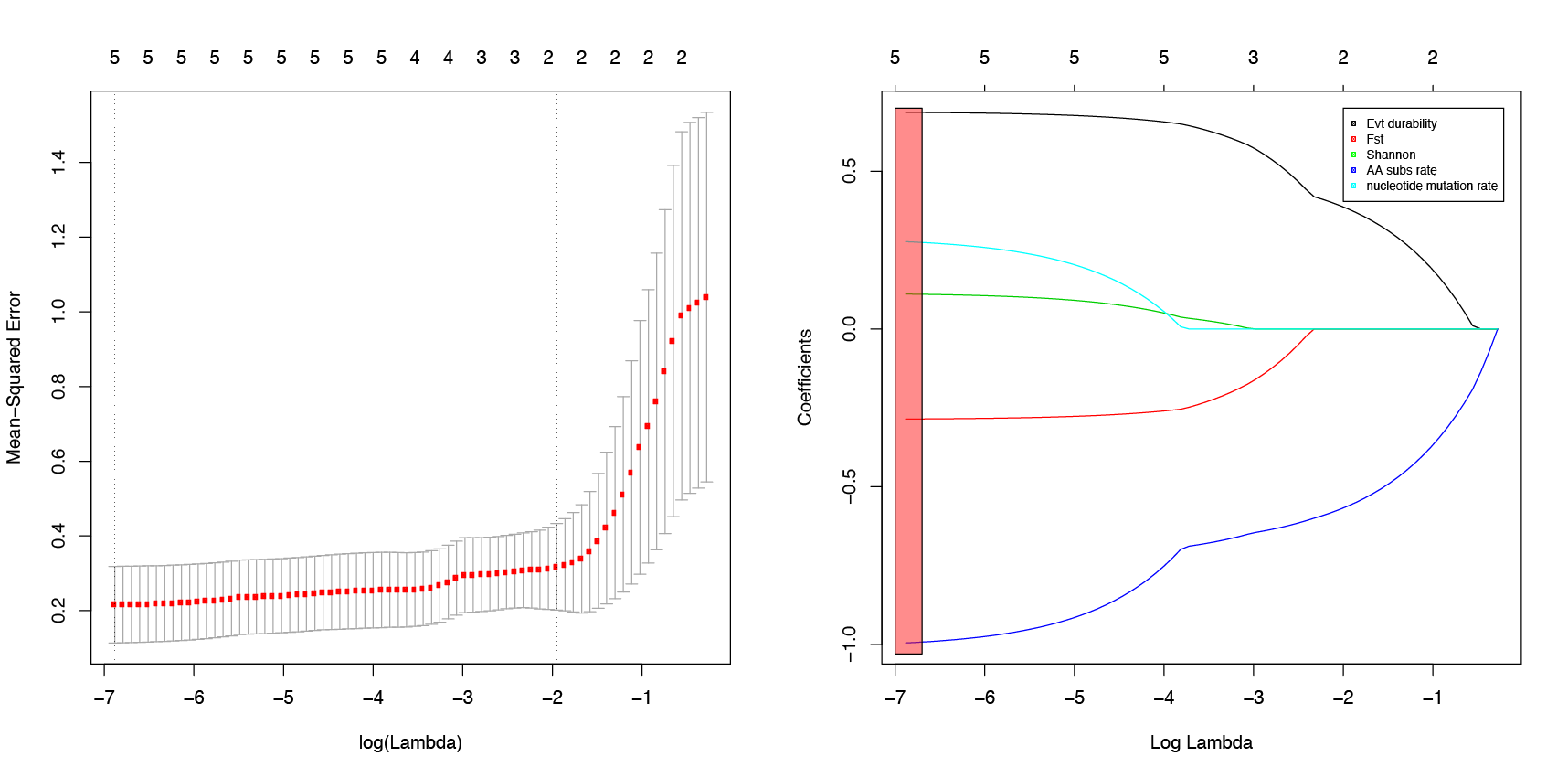

Supplement: Figure S10 — Influence of shrinkage on coefficient values. (Left) Mean-squarred error according the log(λ). Optimal value is for log(λ) = −6.88. (Right) Coefficient values for different levels of log(λ); shaded area represents coefficients for the optimal value of log(λ). (TIF) [file pbio.1001931.s010.tif]

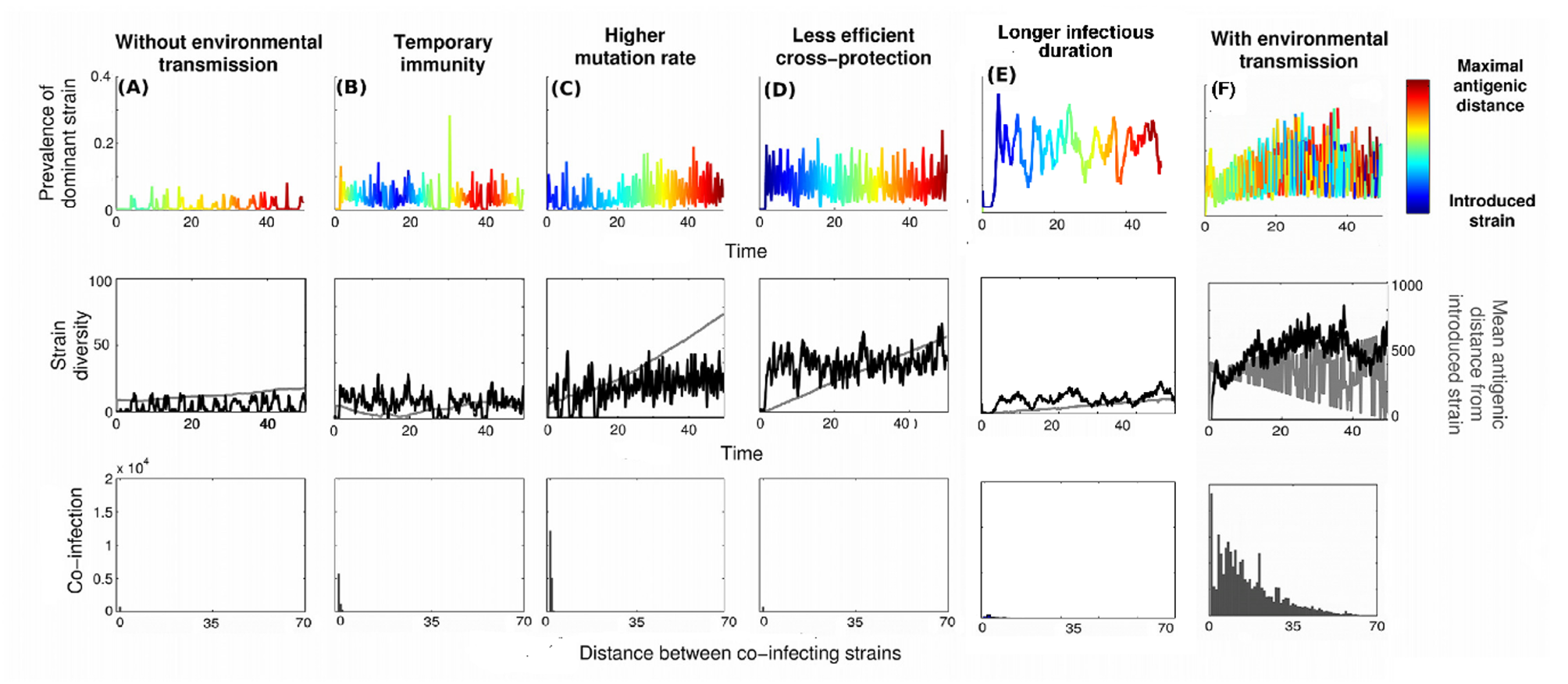

Supplement: Figure S11 — Phylodynamics simulated with different hypotheses than environmental transmission. Parameters are the same as in Figure 3 (A) without environmental transmission. (B) A temporary immunity of 6 mo is assumed. (C) Mutation rate is set at 2.10−5 per base per day on 12,000 bases (assuming the whole genome is coding for antigenic variation) instead of 2,000 (assuming that only mutation on HA is coding). (D) Less efficient cross-protection (changing the cross-immunity parameter d = 1, see eqn. S1 in Text S1). (E) Longer infectious period (20 d) by keeping R0 of direct transmission constant. (F) With environmental transmission. Co-infection patterns are depicted by the absolute numbers of co-infection events in order to complete the figures shown in the main text, where number of co-infections are scaled by host population size. (TIF) [file pbio.1001931.s011.tif]

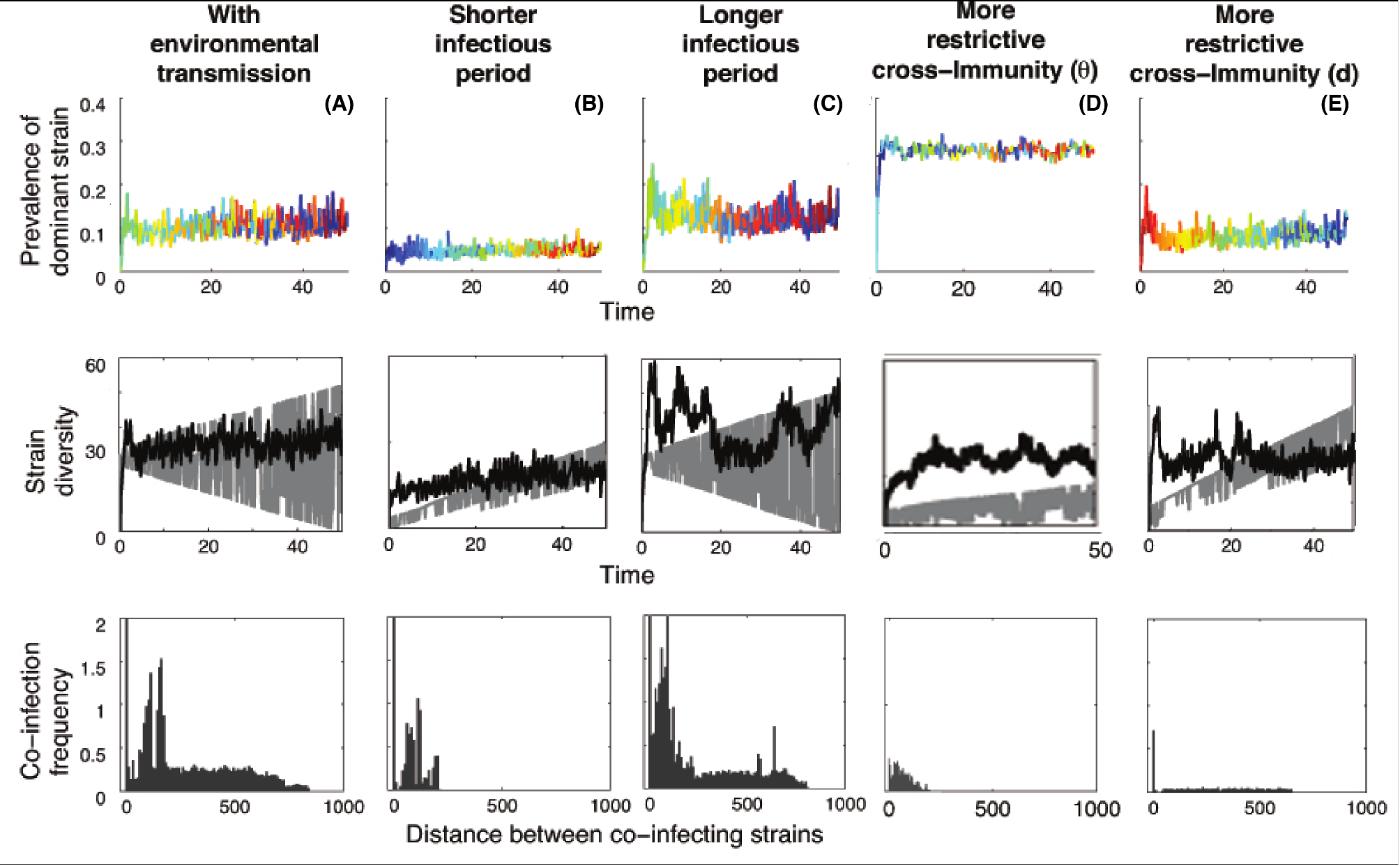

Supplement: Figure S12 — Phylodynamics simulated with environmental transmission and different values for key parameters. Parameters are the same as in Figure 3 with environmental transmission. (B) Infectious period is set at 3 d. (C) Infectious period is set at 7 d. (D) Parameter θ (see eqn S1 in Text S1) is set at 0.5 instead of 0.7. (E) Parameter d (see eqn S1 in Text S1) is set at 5 instead of 3. Co-infection patterns are depicted by the absolute number of co-infection events in order to complete the figures shown in the main text, where number of co-infections are scaled by host population size. (TIF) [file pbio.1001931.s012.tif]

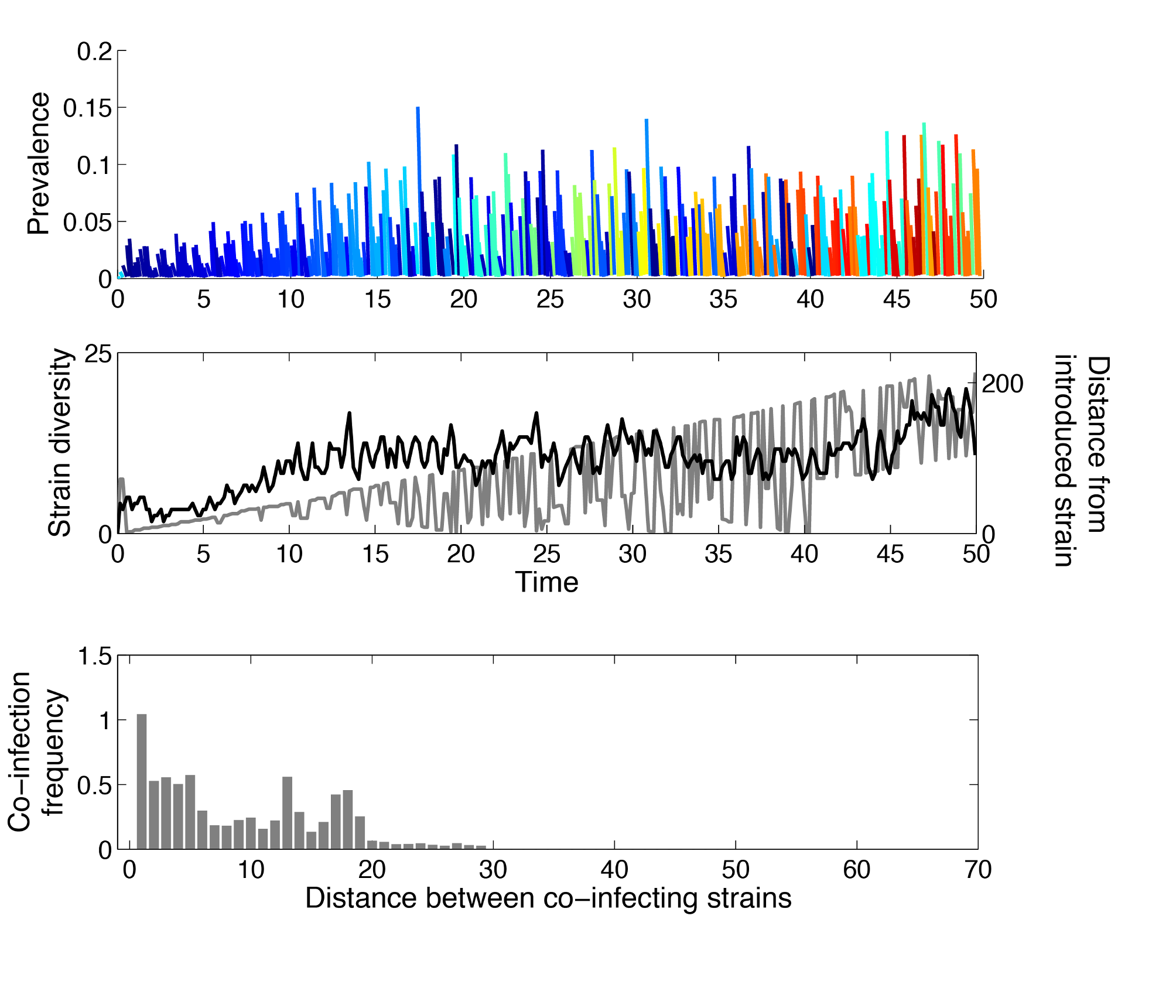

Supplement: Figure S13 — Comparable simulation to Figure 3F, but with the lower values of mutation rate, as assumed in [5]. (TIF) [file pbio.1001931.s013.tif]

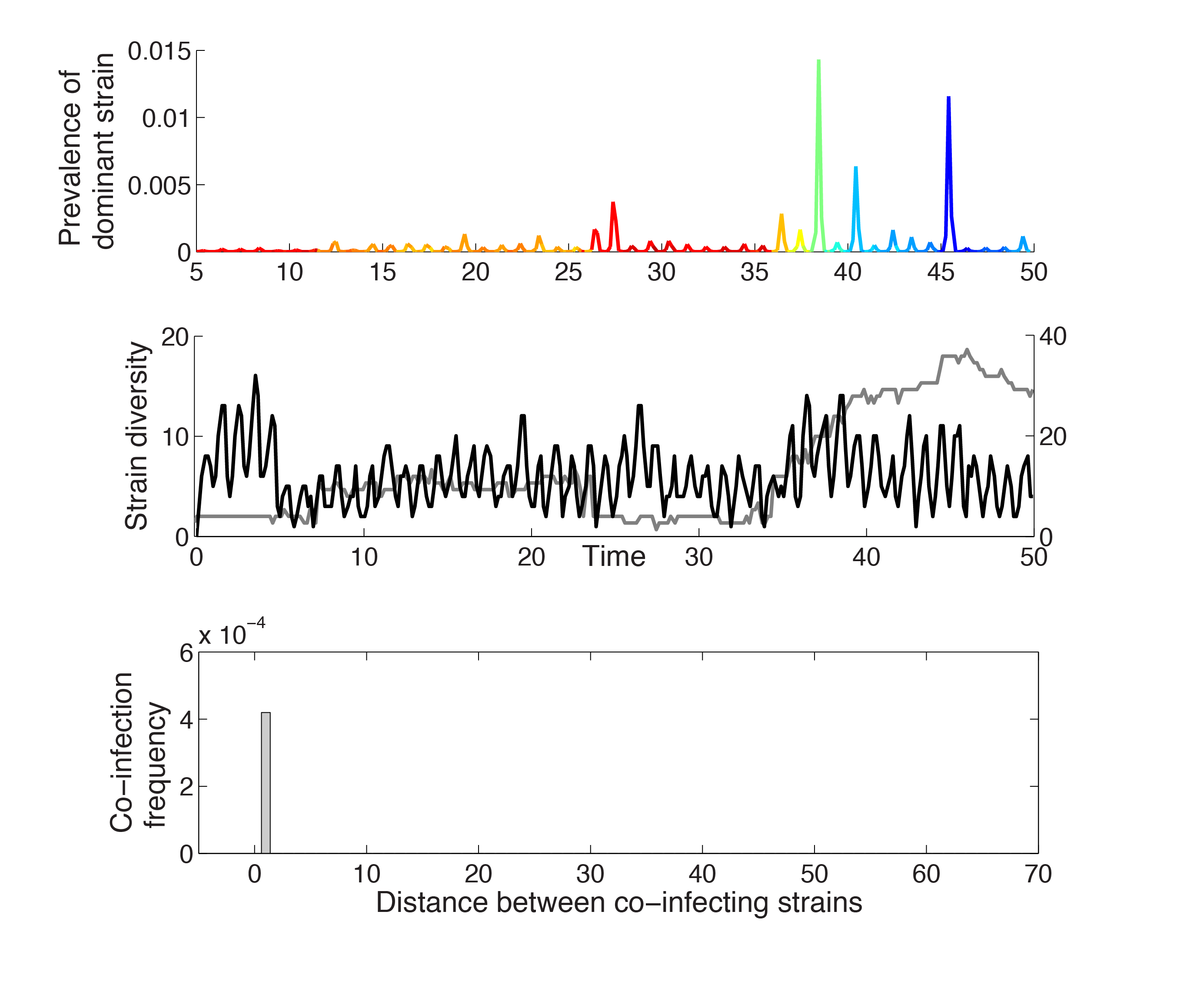

Supplement: Figure S14 — Same simulation as for Figure 3A,D, but with 5 million individuals instead of 1 million. (TIF) [file pbio.1001931.s014.tif]

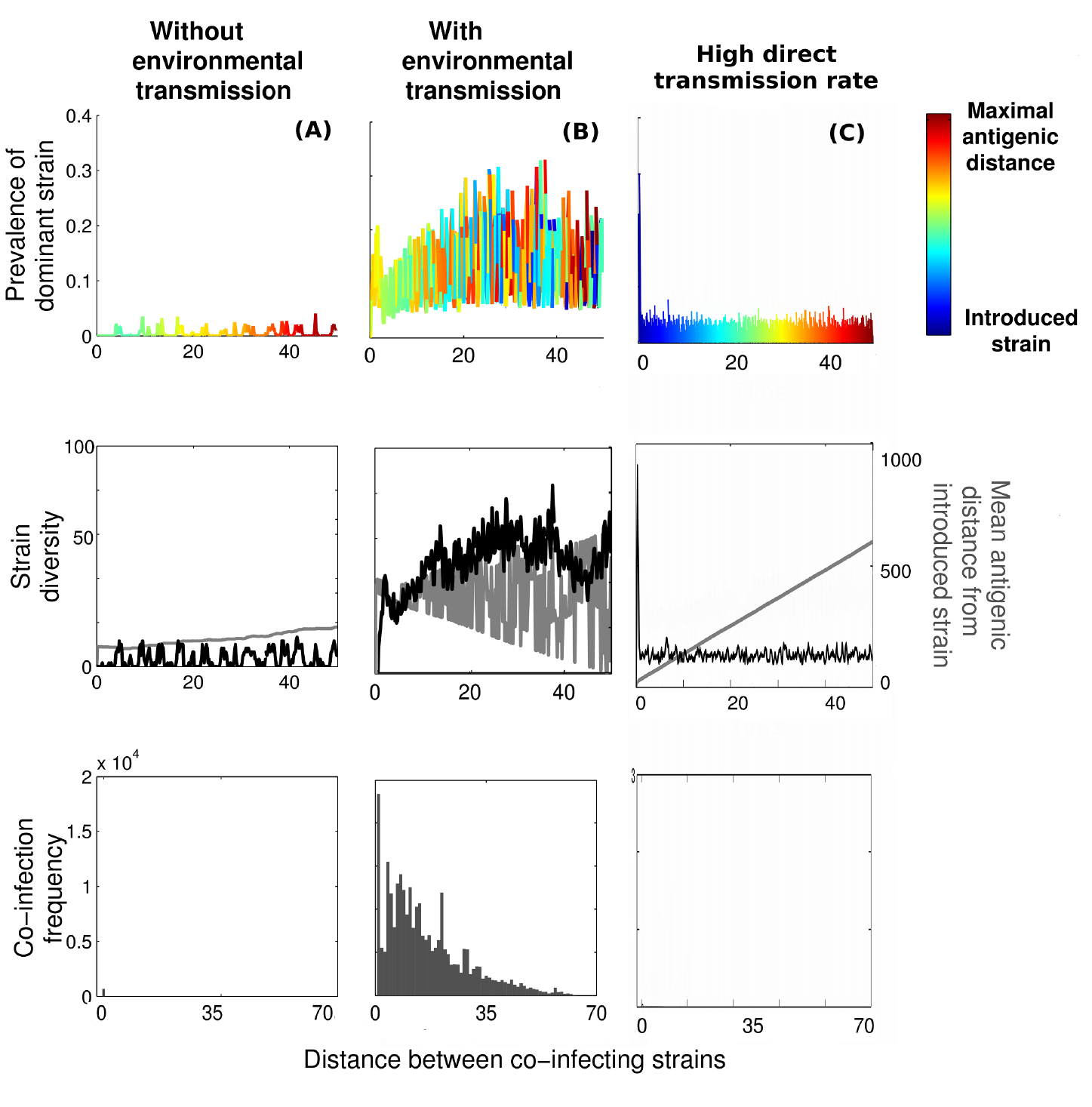

Supplement: Figure S15 — Phylodynamics pattern generated for avian configuration (A) without and (B) with environmental transmission. (C) Considering direct transmission with an increased transmission rate () does not generate a notable increase in strain diversity. (TIF) [file pbio.1001931.s015.tif]

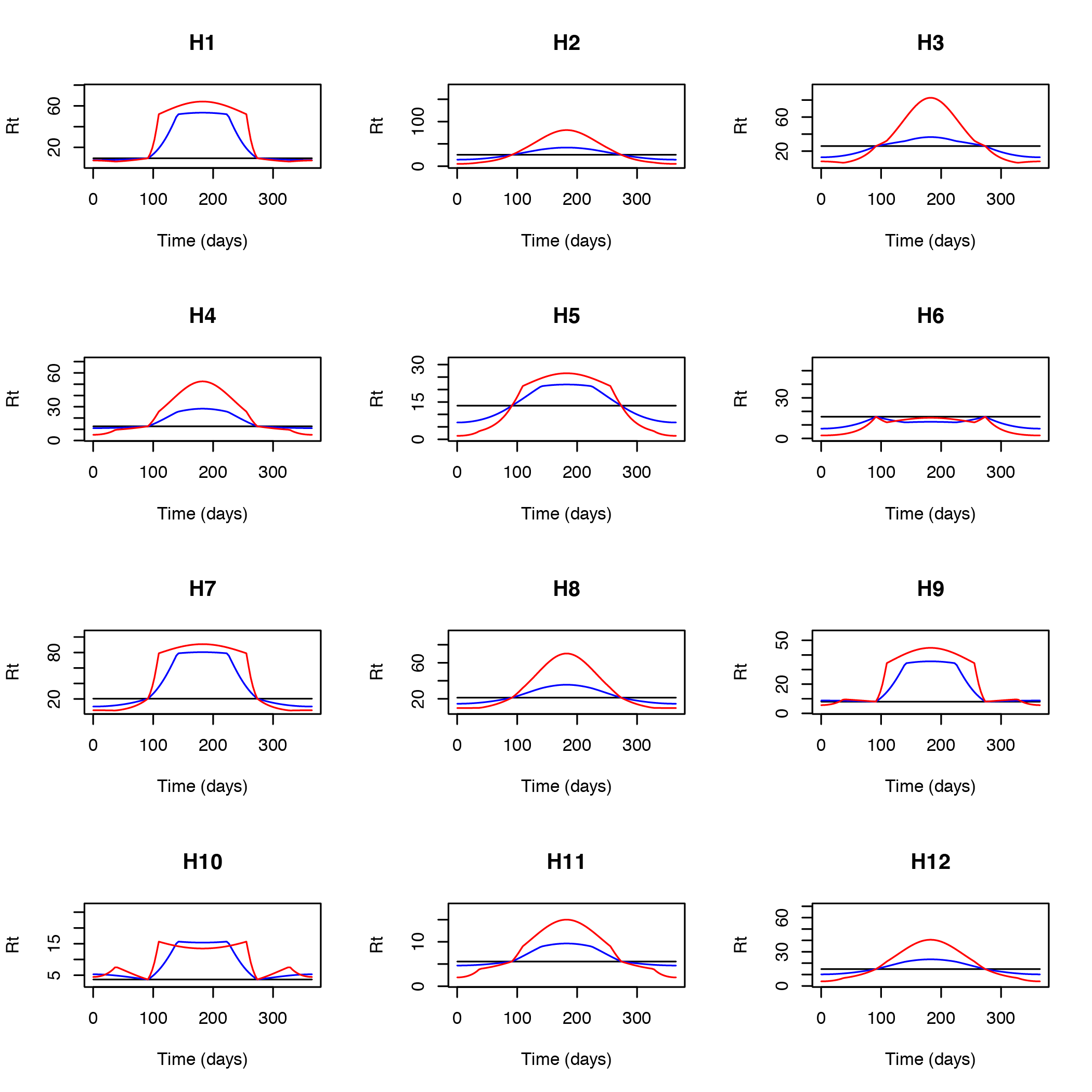

Supplement: Figure S16 — Environmental durabilities estimated when temperature fluctuates through a cosinus function (with an average of 20 degrees) with an amplitude of 0 (black lines), 0.2 (blue lines), and 0.5 (red lines). (TIF) [file pbio.1001931.s016.tif]
