# Supplementary material for: Adaptive Evolution and Environmental Durability Jointly Structure Phylodynamic Patterns in Avian Influenza Viruses
Source: PLoS Biol. 2014 Aug 12;12(8):e1001931. doi: 10.1371/journal.pbio.1001931 (PMC4130664; doi:10.1371/journal.pbio.1001931)
Supplement: Text S1 — Model description and validation, and supplementary statistical and theoretical analyses. (PDF) [file pbio.1001931.s022.pdf]

Adaptive evolution and environmental durability jointly structure phylodynamic patterns in avian influenza viruses

Supplementary materials

Benjamin Roche[1,2], John M. Drake[3], Justin Brown [4], David Stallknecht[4], Trevor Bedford[1,5] and Pejman Rohani[1,6,7]

[1]Department of Ecology and Evolutionary Biology, University of Michigan, Ann Arbor, MI 48109, USA

[2]UMI IRD/UMPC 209 - UMMISCO, 93143, Bondy, France

[3]Odum School of Ecology, University of Georgia, Athens, GA 30602, USA

[4]The Southeastern Cooperative Wildlife Disease Study, Department of Population Health, College of Veterinary Medicine, University of Georgia, Athens, Georgia 30602, USA

[5]Vaccine and Infectious Disease Division, Fred Hutchinson Cancer Research Center, Seattle, WA

98109, USA

[6]Center for the Study of Complex Systems, University of Michigan, Ann Arbor, MI 48109 USA

[7]Fogarty International Center, National Institutes of Health, Bethesda, MD 20892, USA

These supplementary materials are organized under five sections. In the first section (S1), we present a conceptualization of our most novel and perhaps surprising empirical finding, depicting the contrasting transmission dynamics of human and avian influenza viruses and their concomitant impacts on virus coexistence and diversity. Section S2 provides all relevant information for the construction of sequence data sets. The third section (S3) documents the central algorithm that underpins our Individual-Based Model, together with parameter values and corresponding references. Here, we also outline our method for reconstructing digital phylogenies. The fourth section (S4) is focused on using the sequence data for testing competing hypotheses to explain contrasting influenza diversity in human and avian hosts. The fifth section (S5) contains a comprehensive sensitivity analysis of our model results and specifically our focal conclusions on the role of environmental transmission in shaping phylodynamics of AIVs.

S1 Conceptualization of our focal hypothesis

The most novel finding of this paper is that strong selective pressure exerted by herd immunity in long-lived host species leads to low viral diversity and a ladder-like phylogenetic tree, whereas short-lived species and indirect transmission chains resulting from an environmental virus reservoir lead to virus coexistence. As quantified in our elastic-net regression analyses, this basic causal story explains a significant part of the dramatic differences in the population genetics of avian and human influenza viruses. In figure S1, we provide a schematic explanation of this thesis.

S2 Data

S2.1 Summary

Our dataset is comprised of epidemiological information for human and avian influenza viruses in North America between 1976 and 2001. For transmission dynamics of human influenza viruses (Figure 1A), we present death rates from Pneumonia and Influenza (P & I), known to be an accurate measure of influenza activity [1]. The subtype dominance (Figure 1C) has been documented through annual sampling conducted by the Center for Disease Control and Prevention (CDC), as reported in [2]. Annual prevalence (Figure 1B) and subtype dominance patterns (Figure 1D) of avian influenza viruses have been described in Alberta, Canada [3], with isolates obtained from ducks. Genetic data contain only wild bird species, especially mallard.

Sequence information contains all full-length sequences, to avoid bias during sequences alignment, recorded in the website http://www.ncbi.nlm.nih.gov/genomes/FLU/Database/multiple.cgi for equine, swine, avian and human influenza viruses between 1976 and 2013 (accessed June 1st 2013). We analyze human influenza sequences only in the town of Memphis, TN, USA, to consider a comparable number of genetic sequences to AIVs. The Bayesian Skyline Plot (BSP) shown in figure 1 is very similar to the BSP estimated in New York City [4], which is assumed to represent the pattern of influenza evolutionary dynamics. The temporal and spatial distribution of avian sequences are given in figure S2 and S3 respectively. Table S1 shows how many sequences have included for

each avian subtypes.

Sequence data from the H8 subtype have been excluded from our analyses because of (i) the paucity of isolates (only 60 sequences are available), and (ii) the high geographic clustering, with 2/3 of sequences from Alaska. Aligned sequences for avian subtypes are available on DataDryad website and can be accessed at the following URL: `http://datadryad.org/review?wfID=30637&token=df513dbc-c74d-4c12-8a50-b781ceeac71d` (or on http://datadryad.org with the following DOI: doi:10.5061/dryad.8ct18).

Finally, the data on subtype-specific environmental durability have been compiled from unpublished data summarized in [5]. The figure S4 shows a summary of these data.

S2.2 Resulting phylogenies

The phylogenetic trees resulting from our avian influenza sequences are detailed in figure S5 and S6.

S3 Model and algorithms

S3.1 Summary of Individual-Based Model

We have developed an Individual-Based Model to capture within-subtype phylodynamics of influenza viruses with multiple transmission routes. Our model permits a dynamic strain space that may become very large during simulations. This model has been extensively validated elsewhere [6]. The main algorithm of the IBM is:

FOR EACH TIME STEP

FOR EACH INDIVIDUAL

FOR EACH STRAIN

i IS THE INDEX OF THE CURRENT STRAIN j IS THE INDEX OF THE CLOSEST STRAIN IN INFECTION HISTORY rateInfection=$\beta(1 + c_{trans} sin(2\pi t))I_i\sigma_{ij}$

ProbabilityInfection=$1 - e^{(-rateInfection\delta_t)}\epsilon_{ij}$

IF RAND() $<$ ProbabilityInfection

ADD CURRENT STRAIN TO NEXT INFECTIOUS STRAINS IN INDIVIDUAL

END

rateInfection=$\frac{\rho}{L}\frac{V_i}{\sum V_j}\frac{V_i}{V_i+\kappa}$

ProbabilityInfection=$1 - e^{(-rateInfection\delta_t)}\epsilon_{ij}$

IF RAND()$<$ProbabilityInfection

ADD CURRENT STRAIN TO NEXT INFECTIOUS STRAINS IN INDIVIDUAL

END

END

END

FOR EACH INDIVIDUAL

FOR EACH INFECTIOUS STRAINS

rateRecovery$=\gamma$

ProbabilityRecovery$=1-e^{(-rateRecovery\delta_t)}$

IF RAND()$<$ProbabilityRecovery

MOVE CURRENT STRAIN FROM INFECTIOUS STRAINS TO STRAIN HISTORY

END

END

MOVE RECENTLY ACQUIRED STRAINS TO INFECTIOUS STRAINS

rateNewOffspring$=b(1+c_{dem}cos(2\pi t))$

probaNewOffspring$=1-e^{(-rateNewOffspring\delta_t)}$

IF RAND()$<$probaNewOffspring

CREATE NEW SUSCEPTIBLE INDIVIDUAL

END

rateDeath$=d$

probaDeath$=1-e^{(-rateDeath\delta_t)}$

IF RAND()<probaDeath

DELETE CURRENT INDIVIDUAL

END

END

FOR EACH STRAIN

rateClearance=$\xi(1 + c_{evt} sin(2\pi t))$

$V_i = \omega I_i/rateClearance + e^{-rateClearancet)}(V_i - (\omega \frac{I_i}{rateClearance}))$

END

where parameters and their values are detailed in table S2.

S3.2 Mutation rate and cross-immunity

Mutation rate and the cross-immunity network are key parameters of influenza evolution. Throughout this study, we have considered mutation rate to be 2.10^{-5}/base/day [11]. Since the length of Hemagglutinin gene is 2 kb [10] and [11] estimated that 80% of mutations are neutral, *i.e.,* yielding no significant antigenic variation, we assume that a new antigenic variant appears, on average, at a rate of $m = 2.10^{-5} \times 2.10^3 \times 0.2 = 0.008$ per day.

Cross-immunity has been suggested to decrease exponentially with antigenic distance [11, 12]. A

suggested functional form [13], consistent with cross-protection estimated in humans [11] and horses [12], is:

$$\epsilon_{ij} = (1 - e^{-\frac{(i-j)^2}{d}})\theta \tag{S1}$$

where i and j denote antigenic variants, d is a shape parameter and θ is the minimal cross-immunity. In this formulation, ϵ represents the probability that an individual recently infected by strain i will be infected with strain j, upon exposure. Hence, ϵ ranges from 0 (full cross-protection) to 1 (full susceptibility). Throughout the manuscript, we assumed $d = 3$ and $\theta = 0.7$.

S3.3 Reconstructing digital phylogenies

To increase the execution speed of our IBM, only non-neutral antigenic changes have been recorded during simulation. After the simulation is complete, we reconstruct both sequences and mutations, in order to analyze simulation output in an identical way to influenza genetic data and construct phylogenies (Figure S7).

The object-oriented nature of our implementation allows us to track the real genealogy between different samples. After model execution, we walk along this tree and add neutral mutations at a rate proportional to the branch length. Functional mutations are always added whenever there is a

change in phenotype. To test this method, we have modified slightly the model to create perfect examples of immune escape and no antigenic evolution to validate that resulting trees are the ones expected (Figure S8).

S3.4 Antigenic dynamics

In order to show that digital phylogenies represent the correct pattern of pathogen evolutionary dynamics, we plot here dynamics of antigenic variants through time for the three configurations described in figure 3 as well as an intermediate situation where environmental durability is lower than in the main text (Figure S9). Both human and avian influenza configurations without environmental transmission exhibit an immune escape pattern (two figures on the left). Including environmental transmission in avian influenza configuration yields a diversification of antigenic variants. The amplitude of this diversification increases with environmental durability (two panels on the right).

S4 Competing hypotheses to explain high avian influenza diversity

In the main text, we show that amino acid substitution rates and environmental transmission are the main contributor to AIV subtype-specific diversity. Here, we complete the picture of elastic-net regression, describe the variables that have been included in our multiple regression analysis and explore the role of other epidemiological and evolutionary parameters that may affect our results on the influence of environmental durability.

S4.1 Shrinkage values of elastic-net regression analysis

To complete the results shown in the main text, figure S10 shows how shrinkage affects coefficient values. In particular, the left panel of figure S10 shows how Mean Squared Error (MSE) changes with shrinkage, demonstrating lowest MSE for $\log(\lambda) = -6.8$. In parallel, the right hand panel demonstrates the coefficients for each effect associated with a particular shrinkage.

S4.2 Variable selection in elastic-net regression

In order to be able to interpret results from elastic-net regression analysis, we describe here the variables that have been included in the analysis as described in the main text (summarized in table S3).

It is worth elaborating that in order to test hypothesis II, we needed to quantify host species diversity per subtype. Because each virus isolate in our data was attributed to a host species, we denoted by n the total number of unique host species from which each AIV subtype was isolated. Then, for each subtype, we calculated the Shannon index of host species diversity as $H = -\log \sum_{i=1}^{n} p_i \ \log \ p_i$, where p_i is the fraction of isolates of the sybtype obtained from host species i.

S4.3 Pathogen life-history traits

The other hypotheses that may affect our results regarding the influence of environmental durability concern pathogen life-history traits and are tested through our theoretical framework. A shorter duration of immunity in avian hosts [10] could lead to more rapid renewal of the susceptible stock, reducing the impact of herd immunity and generating higher pathogen diversity (hypothesis I). Similarly, a higher mutation rate (hypothesis IV) is expected to accelerate immune escape and reducing the impact of evolutionary bottlenecks. Finally, one can suppose that human and avian immune systems are different enough to explain itself the difference of genetic diversity.

Starting from the baseline scenario of direct transmission only (ie, in the absence of environmental transmission; Figure S11A), our theoretical results show that reducing the mean duration of immunity (Figure S11B), increasing the mutation rate (Figure S11C), reducing the extent of cross-immunity (Figure S11D, $d = 1$, see eqn. S1) or substantially increasing the mean infectious period (Figure S11E) cannot produce and maintain strain diversity similar to what is observed with environmental transmission (Figure S11F). It is worth highlighting that while high mutation rate and reduced cross-immunity can lead to somewhat higher standing genetic diversity, these hypotheses nevertheless lead to a pronounced pattern of immune escape, in contrast to the the broad strain coexistence produced by model output that incorporates environmental transmission and empirical observations.

S5 Sensitivity

In this section, we first focus on the sensitivity of our theoretical findings to changes in assumed parameter values. Then, we quantify the sensitivity of our data analysis to show the range of physical characteristics where our results remain valid.

S5.1 Model sensitivity

We start with the baseline scenario (Figs. S12A) representing avian influenza virus configuration with environmental transmission. We then examined the impacts of systematic variation in key parameters: (i) a shorter infectious period (Figure S12B), a longer infectious period (Figure S12C), (iii) more restrictive cross-immunity resulting from reduced protection, θ (Figure S12D) or (iv) more restrictive cross-protection through reduced antigenic escape with distance, d (Figure S12E). While these changes to parameterization clearly affect the detailed phylodynamic picture, they do not impact our qualitative conclusions regarding viral coexistence with environmental transmission.

We point out that mutation rate used here is significantly higher than that estimated in previous studies [2, 11]. Running the same simulations than for the figure 3F with this lower mutation rate leads to similar results (Figure S13).

Finally, it is important to highlight that results for human influenza (Figure 3A,D,G,J) remain qualitatively similar when bigger population size is considered (Figure S14)

S5.2 Similarity between high β and environmental transmission

Here, we demonstrate that environmental transmission is not functionally equivalent to an increase in direct transmission. Figure S15 shows that increasing β by an order of magnitude leads to a modest increase in strain diversity, but the immune escape dynamics is still noticeable. In contrast to the inclusion of environmental transmission, simply increasing β does not lead to the level of strain diversification shown in figure S9.

S5.3 Variability in simulations

For the sake of readability, each figure shown in the main text displays only a single simulation. In the following table (table S4), we show that different stochastic replicates of our model show very little variation, indicating that the results presented in the main text are representative.

S5.4 Robustness of multiple regression analyses

In this section, we analyze the robustness of our multiple regression analysis according to different temperature values. In the main text, we shown the configuration where the average water temperature is 20 degrees without any seasonality. To introduce seasonality, we mimic seasonal fluctuations in temperature through a cosinus function with different amplitude. To calculate the durability of temperature that has not been experimentally measured, we take advantage of the recent findings of Handel et al. [14] and apply exponential regression between the closest temperatures present in the experimental settings (figure S16).

We then applied the multiple regression analyses on the mean environmental durability according to the amplitude of temperature seasonality. Table S5 shows that strength of selection (estimated by amino acid substitution rates) and environmental durability remain the main factors of genetic diversity.

References

[1] C. Viboud, et al., Science **312**(5772), 447 (2006).

[2] N. Ferguson, A. Galvani, R. Bush, Nature **422**(6930), 428 (2003).

[3] S. Krauss, et al., Vector Borne Zoonotic Dis **4**(3), 177 (2004).

[4] A. Rambaut, et al., Nature **453**(7195), 615 (2008).

[5] J. D. Brown, G. Goekjian, R. Poulson, S. Valeika, D. E. Stallknecht, Vet Microbiol **136**(1-2), 20 (2009).

[6] B. Roche, J. M. Drake, P. Rohani, BMC Bioinformatics **12**, 72 (2011).

[7] D. C. Bennett, M. R. Hughes, J Exp Biol **206**(Pt 18), 3273 (2003).

[8] B. Roche, et al., Infect. Genet. Evol. **9**, 800 (2009).

[9] P. Rohani, R. Breban, D. E. Stallknecht, J. M. Drake, P. Natl. Acad. Sci. U. S. A. **106(25)**, 10365 (2009).

[10] R. G. Webster, W. J. Bean, O. T. Gorman, T. M. Chambers, Y. Kawaoka, Microbiol. Rev. **56**(1), 152 (1992).

[11] K. Koelle, S. Cobey, B. T. Grenfell, M. Pascual, Science **314**, 1898 (2006).

[12] A. W. Park, et al., Science **326**(5953), 726 (2009).

[13] J. R. Gog, B. T. Grenfell, Proc. Natl. Acad. Sci. U. S. A. **99**(26), 17209 (2002).

[14] A. Handel, J. Brown, D. Stallknecht, P. Rohani PLoS Comput Biol. **9**(3), e1002989 (2013)
